# Plant lamin-like proteins mediate chromatin tethering at the nuclear periphery

**DOI:** 10.1186/s13059-019-1694-3

**Published:** 2019-04-30

**Authors:** Bo Hu, Nan Wang, Xiuli Bi, Ezgi Süheyla Karaaslan, Anna-Lena Weber, Wangsheng Zhu, Kenneth Wayne Berendzen, Chang Liu

**Affiliations:** 1 Center for Plant Molecular Biology (ZMBP), University of Tübingen, Auf der Morgenstelle 32, 72076 Tübingen, Germany; 2 Department of Molecular Biology, Max Planck Institute for Developmental Biology, 72076 Tübingen, Germany

**Keywords:** *Arabidopsis*, Lamin, Chromatin structure, Nuclear periphery

## Abstract

**Background:**

The nuclear envelope not only serves as a physical barrier separating nuclear content from the cytoplasm but also plays critical roles in modulating the three-dimensional organization of genomic DNA. For both plants and animals, the nuclear periphery is a functional compartment enriched with heterochromatin. To date, how plants manage to selectively tether chromatin at the nuclear periphery is unclear.

**Results:**

By conducting dual-color fluorescence in situ hybridization experiments on 2C nuclei, we show that in *Arabidopsis thaliana*, specific chromatin positioning at the nuclear periphery requires plant lamin-like proteins CROWDED NUCLEI 1 (CRWN1), CRWN4, and DNA methylation in CHG and CHH contexts. With chromosome painting and Hi-C analyses, we show global attenuation of spatial chromatin compartmentalization and chromatin positioning patterns at the nuclear periphery in both the crwn1 and crwn4 mutants. Furthermore, ChIP-seq analysis indicates that CRWN1 directly interacts with chromatin domains localized at the nuclear periphery, which mainly contains non-accessible chromatin.

**Conclusions:**

In summary, we conclude that CRWN1 is a key component of the lamina-chromatin network in plants. It is functionally equivalent to animal lamins, playing critical roles in modulating patterns of chromatin positioning at the nuclear periphery.

**Electronic supplementary material:**

The online version of this article (10.1186/s13059-019-1694-3) contains supplementary material, which is available to authorized users.

## Background

In eukaryotes, genomic DNA in interphase nuclei shows non-random distribution patterns with respect to sub-nuclear space including the nuclear periphery (NP). In metazoans, it is known that the formation of chromatin-NP interaction network is an intrinsic part of establishing interphase chromosome topology and appropriate transcription regulation at the NP (reviewed recently by [1]). At the molecular level, specific positioning of genomic regions named lamina-associated domains (LADs) at the NP requires the repressive histone mark H3K9 methylation, the lamina network, and many bridging proteins that connect chromatin to the lamina [1–3]. The nuclear lamina is a meshwork layer underneath the nuclear envelope, consisting of lamin and lamin-associated membrane proteins, which collectively provide mechanical support for nuclear architecture [4].

Lamin proteins are only found in metazoans; on the other hand, early biochemical and microscopic studies in plants indicated the presence of plant lamina [5–7]. To date, three types of plant-specific proteins, localized preferentially or exclusively at the inner nuclear membrane (INM), have been listed as candidate lamin analogs. In *Arabidopsis thaliana*, the *CROWDED NUCLEI 1-4* (*CRWN1-4*) genes encode proteins belonging to the nuclear matrix constituent protein (NMCP) family members which are distributed widely across land plants [8–11]. Among the four *Arabidopsis* CRWN proteins, CRWN1 and CRWN4 exhibit predominant localization at the INM and nuclei of *CRWN1* or *CRWN4* loss-of-function mutants show reduced size and increased sphericity [12, 13]. In addition, CRWN1 and CRWN4 have redundant functions with other CRWNs in modulating chromocenter integrity and heterochromatin condensation [14, 15]. Furthermore, CRWN1 is required for positioning *Arabidopsis* chromocenters (consist of centromeric and pericentromeric heterochromatin) at the NP [15]. The second class of candidate plant lamins, whose member is named KAKU4 in *Arabidopsis*, is specific to angiosperms [16, 17]. Similar to *crwn* mutants, *kaku4* mutant plants form spherical nuclei with reduced nuclear size [17]. At the NP, KAKU4 can physically interact with CRWN1 and CRWN4, and together they can deform the nuclear envelope in a dose-dependent manner [17]. A recent study discovered *Arabidopsis* plant nuclear envelope-associated proteins (NEAPs) as the third class of candidate plant lamins [18]. NEAPs are anchored at the INM with trans-membrane domains, interacting with members of the LInker of the Nucleoskeleton and Cytoskeleton (LINC) complex [18].

A growing number of studies in *Arabidopsis* have suggested roles of plant lamins in regulating chromatin organization and gene expression at the NP apart from being structural proteins that control plant nuclear morphology (reviewed recently in [19, 20]). Firstly, as mentioned above, changes in chromatin organization have been observed in some plant lamin mutants. For instance, genome-wide chromatin-chromatin interaction analyses on *crwn1* and *crwn4* plants revealed alterations in contact strength among pericentromeric chromatin on different chromosomes [21]. Secondly, some plant lamins have been shown to interact with transcriptional regulators such as the interaction between CRWN1 and NAC transcription factor NTL9, or between CRWN1 and Polycomb Repressive Complex 2-associated factor PWO1, or also NEAP1 and a bZIP transcription factor bZIP18 [18, 22, 23]. Likewise, *Arabidopsis* transcription factors MYB3 and BIM1 have been shown to interact with carrot NMCP proteins in a heterologous screen [24].

Perinuclear localization of *Arabidopsis* chromatin is not random. It is well known that chromocenters show preferential association with nuclear boundaries [25, 26]. In addition, by using a Restriction Enzyme-Mediated Chromatin Immunoprecipitation (RE-ChIP) method, we have recently showed that intermittent chromatin regions at *Arabidopsis* chromosome arms tend to be specifically positioned at the NP (referred to as NP-enriched domains) [27]. In the RE-ChIP method, a fusion protein of nucleoporin NUP1 and green fluorescent protein, which was specifically localized at the nuclear envelope, was used as a bait to pull down chromatin at its proximity. NP-enriched domains are enriched with transposons and inactive protein-coding genes [27]. These spatial chromatin distribution patterns suggest that the plant NP is a functional sub-compartment, where transcriptional regulation is different from that in the nuclear interior. However, as the NP-enriched domains were identified with an engineered bait protein, the RE-ChIP approach did not reveal information about how plants achieve such spatial chromatin positioning. In this study, we show that *CRWN1*, *CRWN4*, and non-CG DNA methylation are involved in regulating chromatin tethering at the NP. In particular, loss of *CRWN1* results in not only loss of specific chromatin distribution at the NP, but also attenuated chromatin compartmentalization on a genomic scale. Notably, CRWN1 directly interacts with repressed chromatin domains that are positioned at the NP. Our results point CRWNs as functional analogs of animal lamins, providing a framework for further in-depth investigation of lamina-chromatin interactions in plants.

## Results

### Comparative genomic loci positioning at the NP with fluorescence in situ hybridization

**Fig. 1:**
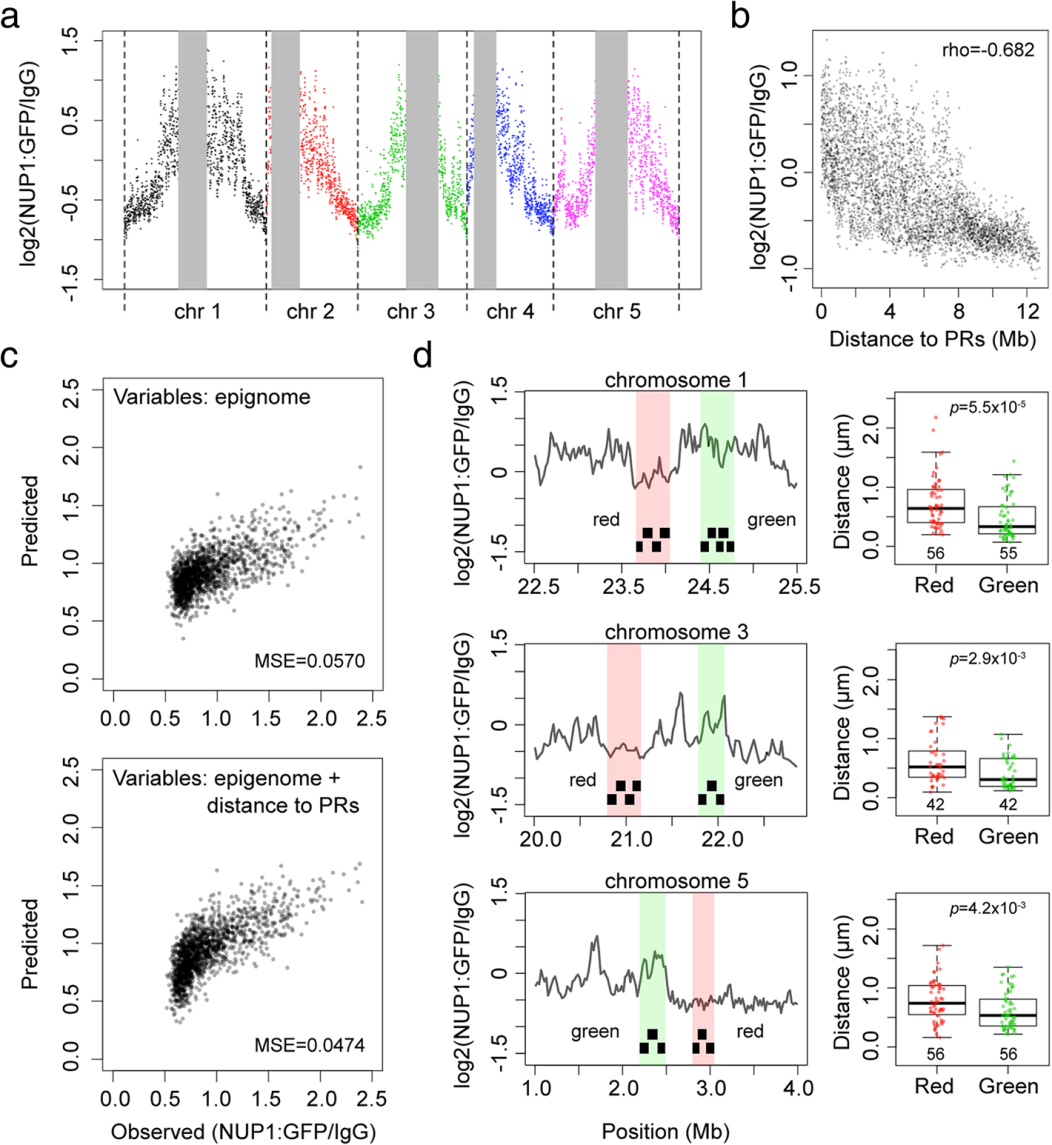
Specific positioning of *Arabidopsis* chromatin at the NP. **a** NUP1:GFP RE-ChIP-seq signals on chromosome arms. The vertical gray bars denote pericentromeric regions (PRs). The RE-ChIP-seq results are from [27], plotted in 20-kb windows. **b** RE-ChIP-seq signals in chromosome arm as a function of distance to PRs. rho denotes Spearman’s rank correlation. **c** Comparison of LASSO (least absolute shrinkage and selection operator) regression models of predicting chromatin-NP interactions. The NUP1:GFP RE-ChIP-seq data [27] of chromosomes 1, 2, and 3 were used for generating regression models, while the data of chromosomes 4 and 5 were used for assessing the fit of the models. The input variables generating the model shown on top were from an integrated *Arabidopsis* epigenetic marks described previously [29], while the model shown at the bottom used genomic distances to the PRs as an additional variable. LASSO regression was performed using the R package “glmnet” with default settings [30]. The “lambda.1se” value, which was determined by tenfold cross-validation, was set as the lambda for model fitting. MSE mean squared error. **d** Probing chromatin localization with FISH. In each row, the plot on the left hand side shows two pools of tiling BAC probes (black segments) designed according to the NUP1:GFP RE-ChIP results [27]. Each pool of probes targets a ~ 300-kb genomic region. Boxplots show distances of the FISH signals from the NP in 2C nuclei. *p* values indicate the Mann-Whitney *U* test results

To understand how plants selectively tether chromatin to the NP, we sought to use fluorescence in situ hybridization (FISH) to identify mutant(s) no longer showing specific perinuclear chromatin anchoring patterns. We applied the following criteria to choose genomic regions for FISH experiments and design probes: firstly, we only selected bacterial artificial chromosomes (BACs) whose signal specificity had been validated [28]. Secondly, the probed genomic regions must be several megabase pairs (Mb) apart from pericentromeric regions (PRs). Thirdly, for each pair of probes, the one hybridized to chromatin preferentially positioned at the NP must be further away from PR compared to the other. The reason that we paid attention to PRs in our FISH probe design was due to possible position effects that passively brought chromatin to the NP. At a chromosomal scale, the closer a locus is to the PR the stronger it tends to be localized to the NP (Fig. 1a, b). Indeed, our regression modeling of chromatin-NP interactions revealed that they were best explained when genomic distances to PRs were included as an additional variable for model fitting (Fig. 1c). As PRs are preferentially localized at the NP, chromatin regions closer to PRs might passively exhibit NP-favored positioning patterns [25, 26].

We designed three pairs of probes targeting regions located on chromosome 1, 3, and 5 (Fig. 1d, left panels and Additional file 2: Table S1) based on our NUP1:GFP RE-ChIP data [27]. Probes of each pair were labeled with different colors: we named those hybridized to NP-enriched chromatin “Green,” which were detected as green fluorescent signals. On the other hand, we used the term “Red” to refer to probes hybridized to genomic regions not enriched at the NP. Besides, only 2C nuclei were collected as input material. It is not known whether endoreduplication can change chromatin positioning at the NP. Nonetheless, changes in chromatin structures in endoreduplicated plant nuclei, like chromatin decondensation, have been reported [31]. Thus, instead of using a mixture of nuclei with different endopolyploidy levels, we selected only 2C nuclei for FISH analyses. Moreover, in this study, unless otherwise stated, all other experiments were restricted to using 2C nuclei collected from aerial parts of 10-day-old seedlings as the input material (see the “Methods” section). In wild-type nuclei, all the three pairs of FISH probes gave results consistent with our NUP1:GFP RE-ChIP data [27]: genomic regions visualized with Green probes had shorter distances to the NP than did those with Red probes (Fig. 1d). Subsequently, we used these FISH probes to unveil whether or not there were changes in perinuclear chromatin anchoring in different plants.

### Specific chromatin positioning at the NP requires plant lamin-like proteins and non-CG DNA methylation

**Fig. 2:**
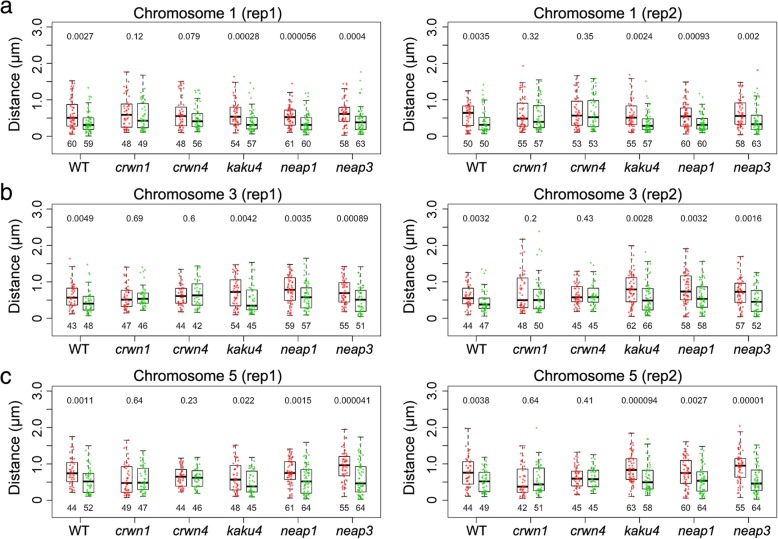
Analyses of FISH signals in *Arabidopsis* lamin-like gene mutants. **a**–**c** Distances of probed genomic regions in chromosome 1 (**a**), 3 (**b**), and 5 (**c**) to the NP are compared in 2C nuclei of various genetic backgrounds. Boxplots with green and red dots denote data of Green and Red probes (same as those shown in Fig. 1d), respectively. For each pair of comparison (boxplots of the same genotype), the *p* value on top indicates the Mann-Whitney *U* test result. WT, wild-type

Functional studies of lamin proteins in various metazoan species, such as *Caenorhabditis elegans*, *Drosophila*, and mouse, have demonstrated their roles of tethering heterochromatin to the NP [32–34]. Thus, by using the three pairs of FISH probes described in the previous section, we sought to test whether chromatin positioning at the NP was affected in plant lamin mutants, namely *crwn1*, *crwn4*, *kaku4*, *neap1*, and *neap3* (Table 1). We used the term “differential localization” to refer to the situation in wild-type nuclei where the Green probes were found closer to the NP than were the Red probes. For all the three pairs of genomic loci, we found differential locations of the probes in *kaku4*, *neap1*, and *neap3*, but not in *crwn1* or *crwn4* nuclei (Fig. 2 and Table 1). *Arabidopsis* CRWN proteins were initially named “LITTLE NUCLEI,” as their loss-of-function mutants had spherical and smaller nuclei [12, 13]. Compared with wild-type nuclei, the changes of FISH signal distribution in *crwn1* and *crwn4* might be attributed to the changes of nuclear morphology. To verify this point, we compared *crwn1*, *crwn4*, and *kaku4* nuclei, in which similar alteration of nuclear morphology had been reported [17]. For 2C nuclei, the differences of nuclear sphericity among wild-type, *crwn1*, *crwn4*, and *kaku4* were not significant (Additional file 1: Figure S1a). The reason of the discrepancy between our observation and previous reports concerning changes of nuclear morphology in the *crwns* and *kaku4* mutants was due to the selection of nuclei that did not undergo any endoreduplication. On the other hand, significant differences in nuclear sphericity could be seen when we compared nuclei with a higher endopolyploidy level (e.g., 8C), which were consistent with the published data [12, 13, 17] (Additional file 1: Figure S1b). Nevertheless, significant changes of nuclear size were found in all the three mutant 2C nuclei, in which the decrease of nuclear volume in *kaku4* tended to be more than that in *crwn1* and *crwn4* (Additional file 1: Figure S1a). Therefore, in *crwn1* and *crwn4* nuclei, the loss of differential positioning of Green and Red probes to the NP was unlikely due to changes in nuclear size.

**Table 1:** Distance measurement of selected genomic loci to the NP (in micrometers) in various candidate lamin mutants

		Chr 1 probes (mean ± sd, *n*)			Chr 3 probes (mean ± sd, *n*)			Chr 5 probes (mean ± sd, *n*)		
		Red	Green	*p*	Red	Green	*p*	Red	Green	*p*
Rep1	Wild-type	0.60 ± 0.41, 60	0.40 ± 0.29, 59	**	0.62 ± 0.34, 43	0.45 ± 0.31, 48	**	0.79 ± 0.37, 44	0.56 ± 0.37, 52	**
	*crwn1*	0.65 ± 0.43, 48	0.55 ± 0.38, 49	ns	0.58 ± 0.32, 47	0.60 ± 0.29, 46	ns	0.59 ± 0.42, 49	0.59 ± 0.35, 47	ns
	*crwn4*	0.62 ± 0.42, 48	0.50 ± 0.33, 56	ns	0.64 ± 0.28, 44	0.69 ± 0.39, 42	ns	0.65 ± 0.26, 44	0.61 ± 0.27, 46	ns
	*kaku4*	0.61 ± 0.35, 54	0.42 ± 0.30, 57	**	0.73 ± 0.41, 54	0.51 ± 0.38, 45	**	0.65 ± 0.42, 48	0.48 ± 0.32, 45	*
	*neap1*	0.55 ± 0.28, 61	0.37 ± 0.27, 60	**	0.79 ± 0.37, 59	0.61 ± 0.37, 57	**	0.78 ± 0.35, 61	0.58 ± 0.38, 64	**
	*neap3*	0.63 ± 0.35, 58	0.44 ± 0.36, 63	**	0.74 ± 0.35, 55	0.52 ± 0.37, 51	**	0.94 ± 0.41, 55	0.61 ± 0.45, 64	**
Rep2	Wild-type	0.59 ± 0.32, 50	0.42 ± 0.34, 50	**	0.60 ± 0.29, 44	0.46 ± 0.30, 47	**	0.77 ± 0.41, 44	0.54 ± 0.31, 49	**
	*crwn1*	0.61 ± 0.44, 55	0.57 ± 0.40, 57	ns	0.72 ± 0.53, 48	0.63 ± 0.49, 50	ns	0.54 ± 0.41, 42	0.56 ± 0.39, 51	ns
	*crwn4*	0.66 ± 0.44, 53	0.64 ± 0.45, 53	ns	0.65 ± 0.29, 45	0.64 ± 0.32, 45	ns	0.64 ± 0.30, 45	0.63 ± 0.28, 45	ns
	*kaku4*	0.59 ± 0.40, 55	0.39 ± 0.28, 57	**	0.79 ± 0.42, 62	0.59 ± 0.43, 66	**	0.87 ± 0.39, 63	0.62 ± 0.41, 58	**
	*neap1*	0.56 ± 0.33, 60	0.38 ± 0.24, 60	**	0.80 ± 0.43, 58	0.60 ± 0.38, 58	**	0.77 ± 0.40, 60	0.57 ± 0.38, 64	**
	*neap3*	0.64 ± 0.39, 58	0.47 ± 0.41, 63	**	0.74 ± 0.39, 57	0.51 ± 0.37, 52	**	0.93 ± 0.42, 55	0.59 ± 0.41, 64	**

*p* value indicates the Mann-Whitney *U* test result. *, *p* < 0.05; **, *p* < 0.01; ns, not significant

**Fig. 3:**
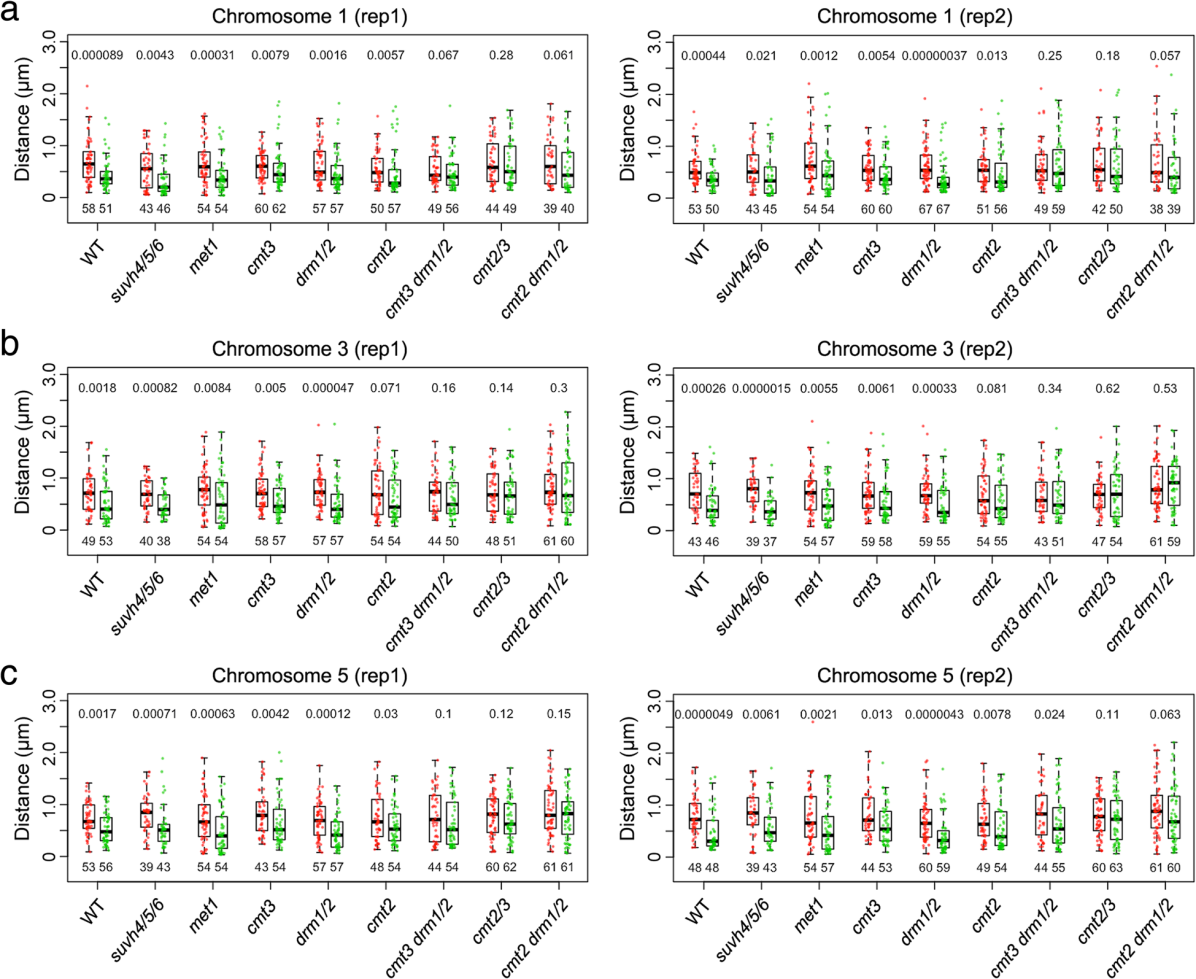
Analyses of FISH signals in *Arabidopsis* epigenetic mutants. **a–c** Distances of probed genomic regions in chromosome 1 (**a**), 3 (**b**), and 5 (**c**) to the NP are compared in 2C nuclei of various genetic backgrounds. Boxplots with green and red dots denote data of Green and Red probes (same as those shown in Fig. 1d), respectively. For each pair of comparison (boxplots of the same genotype), the *p* value on top indicates the Mann-Whitney *U* test result. WT, wild-type

In our previous study, we showed that the localization of *Arabidopsis* transposable elements (TEs) in the nucleus is correlated with the type of CHH (H stands for A, C, or T) DNA methylation pathway [27]. In *Arabidopsis*, CHH DNA methylation is controlled by DOMAINS REARRANGED METHYLASE 1/2 (DRM1/2) and CHROMOMETHYLASE 2 (CMT2), which are the key enzymes in RNA-directed DNA methylation (RdDM) and RdDM-independent pathways, respectively (see reviews by [35, 36]). Specifically, long TEs (> 1 kb) showing preferential localization at the NP lose more CHH methylation in the *cmt2* mutant than do TEs localized in the nuclear interior [27]. This observation prompted us to ask whether CHH methylation contributed to tethering of plant chromatin at the NP. Interestingly, with the same FISH approach described above, we found that the distance distribution patterns of probe pairs were differentially affected in the *cmt2* mutant: the Green and Red probe pairs of chromosome 1 and 5 showed significantly different distance distributions to the NP, while the pair of chromosome 3 could not be differentiated (Fig. 3 and Table 2). For the *drm1 drm2* double mutants, the Green probes of each probe pair showed shorter distance to the NP than the Red probes. In parallel, we tested whether loss of CMT3, the main CHG methyltransferase, could result in changes of FISH signal distributions. Similar to the results obtained from wild-type nuclei, all pairs of probes showed differential distance to the NP in *cmt3* (Fig. 3 and Table 2). Interestingly, for higher-order CHG and CHH methylation mutants (*cmt3 drm1 drm2*, *cmt2 cmt3*, and *cmt2 drm1 drm2*), the distance of Green and Red probes to the NP could no longer be differentiated. On the contrary, in *met1* mutants that lost CG methylation, differential location of Green and Red probes at the NP could still be observed (Fig. 3 and Table 2). Collectively, these results suggest that non-CG DNA methylation pathways are redundantly required for specific tethering chromatin at the NP in plants.

**Table 2:** Distance measurement of selected genomic loci to the NP (in micrometers) in various epigenetic mutants

		Chr 1 probes (mean ± sd, *n*)			Chr 3 probes (mean ± sd, *n*)			Chr 5 probes (mean ± sd, *n*)		
		Red	Green	*p*	Red	Green	*p*	Red	Green	*p*
Rep1	Wild-type	0.71 ± 0.42, 58	0.45 ± 0.31, 51	**	0.72 ± 0.37, 49	0.51 ± 0.37, 53	**	0.73 ± 0.30, 53	0.55 ± 0.30, 56	**
	*suvh4/5/6*	0.57 ± 0.40, 43	0.35 ± 0.33, 46	**	0.71 ± 0.30, 40	0.49 ± 0.25, 38	**	0.80 ± 0.37, 39	0.56 ± 0.40, 43	**
	*met1*	0.68 ± 0.43, 54	0.43 ± 0.33, 54	**	0.78 ± 0.43, 54	0.58 ± 0.49, 54	**	0.74 ± 0.44, 54	0.48 ± 0.41, 54	**
	*cmt3*	0.61 ± 0.26, 60	0.55 ± 0.39, 62	**	0.73 ± 0.36, 58	0.57 ± 0.31, 57	**	0.84 ± 0.43, 43	0.65 ± 0.45, 54	**
	*drm1/2*	0.66 ± 0.38, 57	0.48 ± 0.36, 57	**	0.75 ± 0.34, 57	0.52 ± 0.37, 57	**	0.70 ± 0.36, 57	0.46 ± 0.32, 57	**
	*cmt2*	0.56 ± 0.33, 50	0.47 ± 0.43, 57	**	0.75 ± 0.48, 54	0.61 ± 0.41, 54	ns	0.76 ± 0.45, 48	0.60 ± 0.37, 54	*
	*cmt3 drm1/2*	0.55 ± 0.29, 49	0.49 ± 0.33, 56	ns	0.71 ± 0.38, 44	0.64 ± 0.40, 50	ns	0.78 ± 0.48, 44	0.65 ± 0.46, 54	ns
	*cmt2/3*	0.68 ± 0.42, 44	0.64 ± 0.44, 49	ns	0.76 ± 0.40, 48	0.69 ± 0.45, 51	ns	0.80 ± 0.40, 60	0.72 ± 0.39, 62	ns
	*cmt2 drm1/2*	0.71 ± 0.48, 39	0.56 ± 0.44, 40	ns	0.84 ± 0.44, 61	0.84 ± 0.58, 60	ns	0.90 ± 0.49, 61	0.79 ± 0.44, 61	ns
Rep2	wild-type	0.58 ± 0.32, 53	0.40 ± 0.22, 50	**	0.78 ± 0.38, 43	0.51 ± 0.36, 46	**	0.81 ± 0.38, 48	0.48 ± 0.39, 48	**
	*suvh4/5/6*	0.57 ± 0.37, 43	0.44 ± 0.42, 45	*	0.79 ± 0.32, 39	0.43 ± 0.26, 37	**	0.84 ± 0.44, 39	0.60 ± 0.41, 43	**
	*met1*	0.77 ± 0.49, 54	0.53 ± 0.49, 54	**	0.75 ± 0.44, 54	0.54 ± 0.39, 57	**	0.80 ± 0.52, 54	0.55 ± 0.47, 57	**
	*cmt3*	0.59 ± 0.30, 60	0.48 ± 0.31, 60	**	0.72 ± 0.40, 59	0.57 ± 0.40, 58	**	0.84 ± 0.45, 44	0.65 ± 0.37, 53	*
	*drm1/2*	0.63 ± 0.36, 67	0.39 ± 0.32, 67	**	0.75 ± 0.37, 59	0.53 ± 0.36, 55	**	0.69 ± 0.39, 60	0.42 ± 0.35, 59	**
	*cmt2*	0.58 ± 0.36, 51	0.49 ± 0.42, 56	*	0.71 ± 0.46, 54	0.59 ± 0.41, 55	ns	0.71 ± 0.40, 49	0.56 ± 0.43, 54	**
	*cmt3 drm1/2*	0.65 ± 0.44, 49	0.65 ± 0.49, 59	ns	0.69 ± 0.40, 43	0.69 ± 0.47, 51	ns	0.83 ± 0.49, 44	0.66 ± 0.50, 55	*
	*cmt2/3*	0.69 ± 0.46, 42	0.65 ± 0.52, 50	ns	0.68 ± 0.33, 47	0.74 ± 0.49, 54	ns	0.81 ± 0.39, 60	0.72 ± 0.44, 63	ns
	*cmt2 drm1/2*	0.71 ± 0.56, 38	0.57 ± 0.51, 39	ns	0.86 ± 0.44, 61	0.87 ± 0.45, 59	ns	0.93 ± 0.52, 61	0.79 ± 0.54, 60	ns

*p* value indicates the Mann-Whitney *U* test result. *, *p* < 0.05; **, *p* < 0.01; ns, not significant

The heterochromatic mark H3K9me is enriched in chromatin localized at the NP in both animals and plants [27, 37, 38]. In *C*. *elegans*, the anchoring of chromatin at the NP requires H3K9 methylation during embryogenesis [39]. *Arabidopsis* SU (VAR)3-9 HOMOLOG 4 (SUVH4), SUVH5 and SUVH6 are H3K9me2 methyltransferases [40, 41]. Surprisingly, we could still find differential positioning of the Green and Red probes in *Arabidopsis suvh4 suvh5 suvh6* triple mutants that lost H3K9me2 extensively (Fig. 3 and Table 2) [42], suggesting that this histone mark in plants is dispensable for chromatin tethering at the NP.

### *crwn1* mutant loses specific chromatin tethering at the NP at a chromosomal scale

**Fig. 4:**
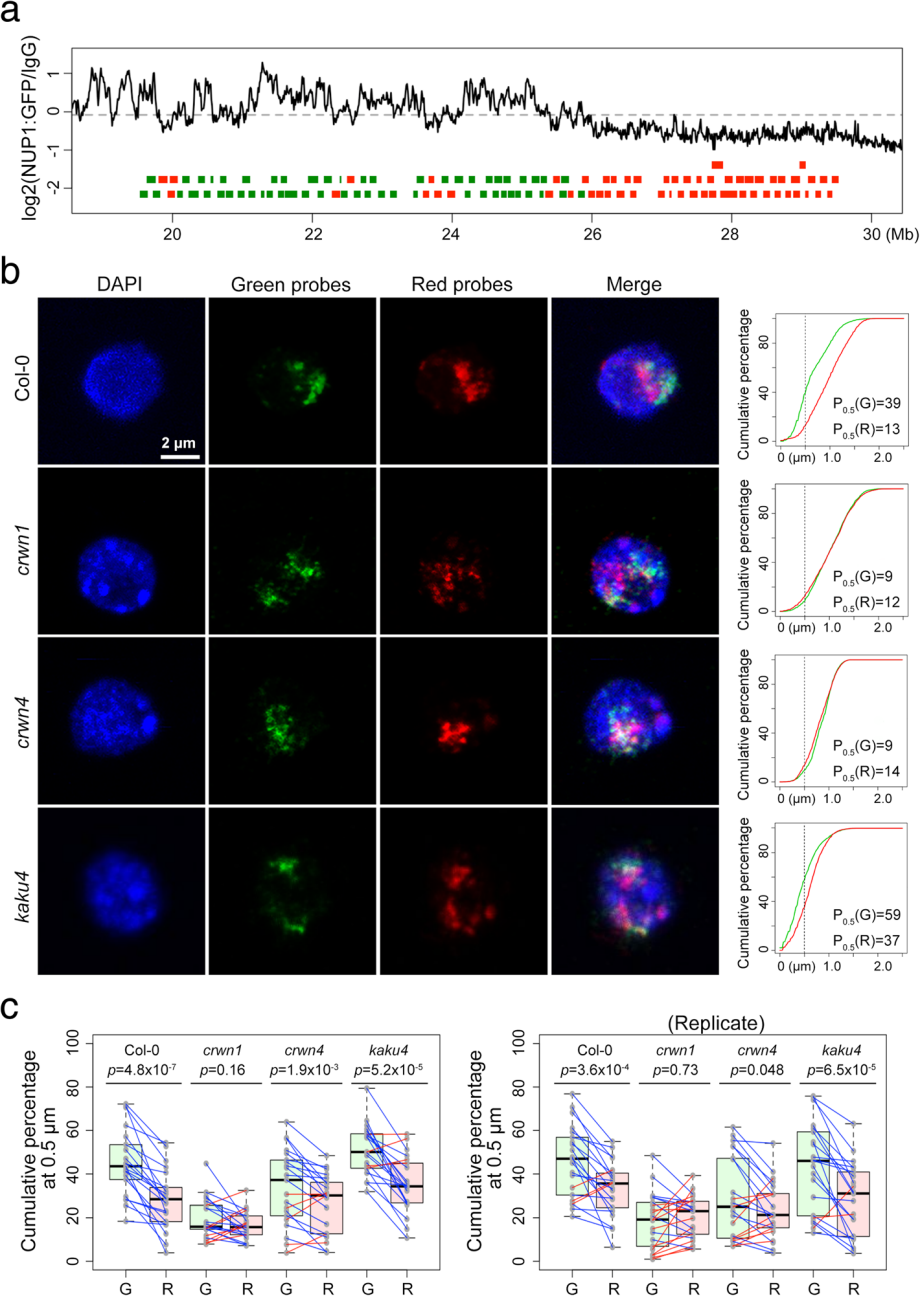
Loss of specific chromatin positioning at the nuclear periphery in *crwn* mutants. **a** Design of dual-color FISH probes targeting a 10-Mb region in chromosome 1 according to NUP1:GFP RE-ChIP results. Green and red rectangles depict BACs. Chromatin regions showing preference of staying closer to the NP (having higher RE-ChIP signals) are labeled as Green probes. **b** Representative confocal images of wild-type and mutant 2C nuclei. The plots on the right hand side show cumulative chromosome painting signal as a function of the distance to the NP. *P*_0.5_ denotes the percentage of signals found within 0.5 μm from the NP. **c** Comparison of chromosome painting signals (*P*_0.5_) in different plants. The *P*_0.5_ values of Green and Red probes of each nucleus are connected by a blue or red line, indicating whether *P*_0.5_(Green) is larger or not, respectively. G and R, Green and Red probes as indicated in **a**, respectively. *p* values mean results of Wilcoxon signed-rank tests

As our FISH experiments on three pairs of loci suggested roles of CRWN proteins in mediating chromatin tethering at the NP (Table 1), we performed a chromosome painting experiment with probes covering a 10-Mb genomic region located at the right arm of chromosome 1 to investigate how CRWNs regulate chromatin positioning at a chromosomal scale (Fig. 4a). This region could be probed with around 100 tiling BACs (Additional file 2: Table S1), among which the color of each BAC (Green or Red) in this chromosome painting experiment was assigned according to our NUP1:GFP RE-ChIP results indicative of how often the region probed by a BAC was close to the NP [27]. For each nucleus, we approximated probe distributions by calculating cumulative probe signals as a function of distance to the NP (Fig. 4b and Additional file 1: Figure S2). We arbitrarily chose *P*_0.5_, which we defined as the percentage of the signal located within 0.5 μm to the NP, as a cutoff to compare how Green and Red probes might be differentially localized. In wild-type 2C nuclei, the *P*_0.5_ values of the Green probes were significantly larger than those of the Red probes (Fig. 4c). Interestingly, a few wild-type nuclei showed an opposite pattern (Fig. 4c, right panel). This cell-to-cell variation implies a scenario similar to that in mammals in which LADs are not identical across a cell population as they are reshuffled upon mitosis [43, 44]. For the *crwn* mutants, both *crwn1* and *crwn4* nuclei lost differential probe positioning patterns to certain extents; in the case of *crwn1* nuclei, the location of Green and Red probes could be no longer differentiated (Fig. 4c), suggesting that CRWN1 protein is the major CRWN required for tethering chromatin at the NP. On the other hand, the differential positioning of Green and Red probes was not affected in the *kaku4* nuclei, which had similar nuclear size and morphology changes to those of the *crwns*, suggesting that plant lamins have divergent functions in terms of regulating chromatin positioning (Fig. 4c).

### *crwn1* mutant exhibits decreased chromatin compartmentalization without changes in local chromatin accessibility

Given the deficiency of specifically tethering chromatin at the NP in *crwn1* plants, we asked whether its chromatin organization adapted an alternative topology by checking genome-wide chromatin interaction patterns with an in situ Hi-C approach [45]. Because only 2C nuclei were collected as the starting material, we modified our in situ Hi-C protocol to cope with such reduced input compared to a typical run, in which all types of nuclei were included (see the “Methods” section). In total, we obtained around 40 million valid Hi-C reads for each genotype with highly correlated replicates (Additional file 1: Figure S3 and Additional file 3: Table S2). At a glance, the Hi-C maps of wild-type, *crwn1*, and *crwn4* plants were highly similar among themselves, suggesting the absence of extensive rewiring of chromatin organization in *crwn* mutants (Additional file 1: Figure S4). Remarkably, the 2C nuclei Hi-C map of wild-type plants highly resembled other published ones, which were based on harvesting nuclei of all ploidy levels [21, 46, 47], indicating that the global chromosome organization patterns are not altered when plant nuclei undergo endoreduplication cycles.

**Fig. 5:**
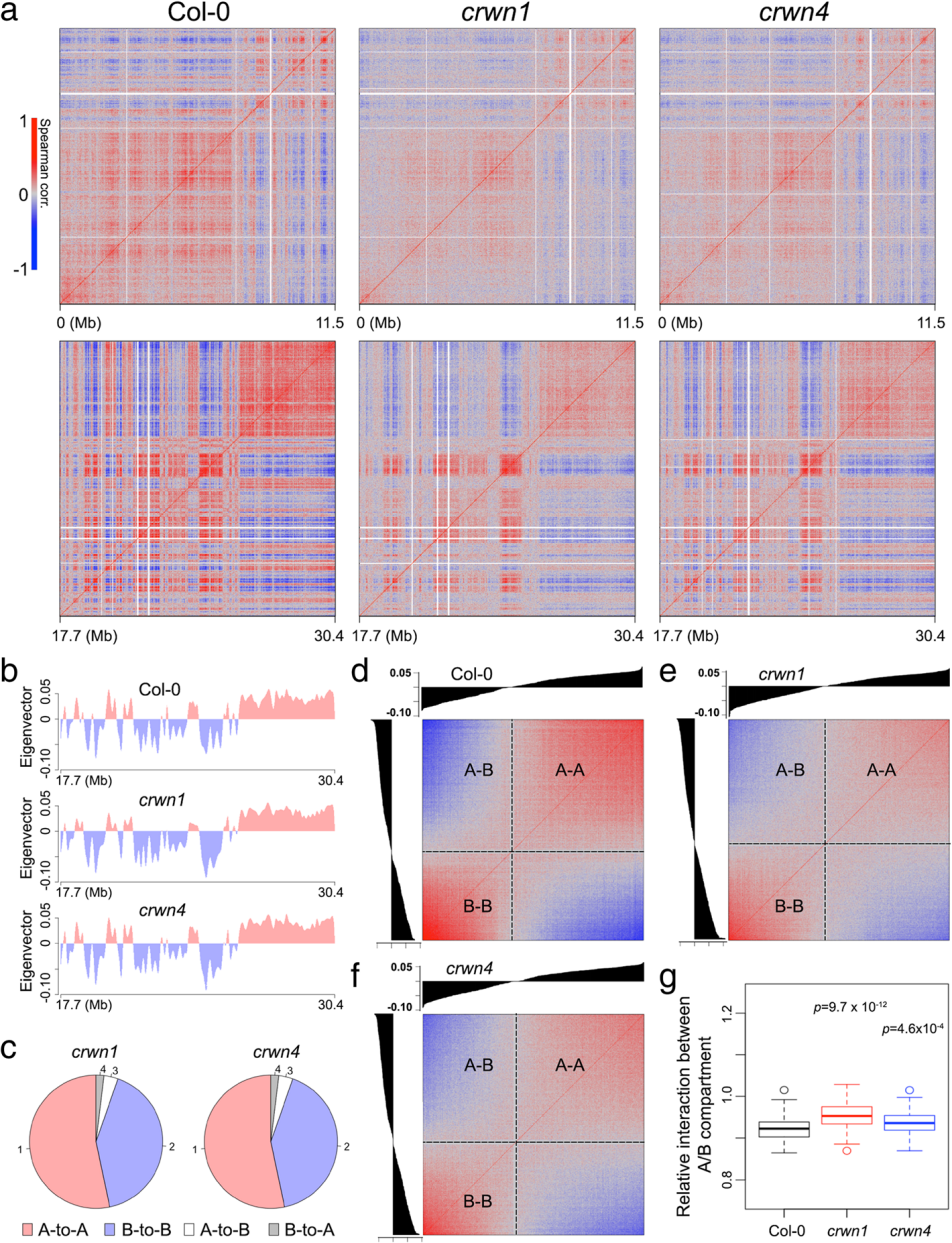
Comparison of chromosomal conformations in 2C nuclei between wild-type and *crwn* mutant plants. **a** Correlation matrices of Hi-C contact maps of chromosome 1 left and right arms. **b** A/B compartment along the chromosome 1 right arm. **c** A/B compartment annotation of chromatin regions of chromosome 1 right arm in *crwn* mutants with respect to that in wild-type plants. **d**–**f** Correlation between pairs of chromatin regions arranged by their eigenvector values shown in **b**. **g** Distance-normalized interaction strengths between A and B compartments relative to the average. Chromatin interactions within 1 Mb are included in the calculation. *p* values indicate the Mann-Whitney *U* test results

However, we found changes in the correlation matrices upon analyzing intra-chromosomal Hi-C maps, in which both the *crwn1* and *crwn4* Hi-C maps exhibited weaker chromatin compartmentalization (Fig. 5a). Because the correlation matrix derived from a Hi-C map is intimately related to how strong interactions/depletions are among chromatin regions, a weakened correlation matrix indicates a less degree of spatial separation of different chromatin compartments. It should be noted here that the chromosome territories did not become completely orderless in *crwn1* or *crwn4* nuclei. Taking the right arm of chromosome 1 as an example, principal component analyses indicated that both the *crwn1* and *crwn4* chromatin could be classified into two spatial compartments (A and B compartments), which were almost the same as those in wild-type plants (Fig. 5b,c). Nonetheless, in these *crwn* mutants, chromatin regions in the same compartment tended to have less positive correlation, while those in different compartments tend to have less negative correlation (Fig. 5d–f). Such decreased chromatin compartmentalization was linked to increased chromatin interactions across different compartments (Fig. 5g), which occurred at a chromosomal scale (Additional file 1: Figure S5).

Additionally, we found that chromatin in *crwn1* and *crwn4* mutants had more frequent inter-chromosomal interactions (Additional file 1: Figure S6). For inter-chromosomal interactions, NP-enriched chromatin domains had decreased interactions among themselves in the *crwn* mutants (Additional file 1: Figure S7). These patterns are consistent with our cytological data showing that NP-enriched chromatin tended to spread in the nuclear interior. Taken together, the increased chromatin interactions between different compartments (e.g., between different chromosomes and between A/B compartments in the same chromosome) indicate that chromatin in *crwn1* and *crwn4* nuclei becomes less ordered.

Observing alterations of chromatin organization in *crwn* mutants prompted us to ask if they were associated with changes in local chromatin structure. To address this question, we employed the Assay for Transposase-Accessible Chromatin using sequencing (ATAC-seq). The ATAC-seq method can reveal distinct euchromatin and heterochromatin accessibility profiles at a chromosomal scale (Additional file 1: Figure S8a). This method can also reveal changes in local chromatin accessibility associated with large-scale rewiring of chromatin structure. For instance, with the ATAC-seq method, we found that the majority of hyper-accessible chromatin regions in *morc6* nuclei were located in pericentromeric regions (Additional file 1: Figure S8b and Additional file 4: Table S3), which was consistent with previous findings showing chromocenter decondensation in this mutant [48]. In addition to comparing chromatin accessibility profiles between wild-type and *crwn1*, we also included *kaku4* nuclei as a control to assess whether potential different ATAC-seq patterns in *crwn1* could be attributed to changes of nuclear architecture per se, because manipulation of gene expression by mechanical forces that change nuclear shape had been shown experimentally [49, 50]. Surprisingly, chromatin accessibility landscape of 2C nuclei in wild-type, *crwn1*, and *kaku4* was highly similar to each other, even for regions that lost perinuclear localization in the *crwn1* mutant (Additional file 1: Figure S9 and Additional file 5: Table S4). We also performed quantitative analysis to call differentially accessible chromatin regions (see the “Methods” section). It turned out that *crwn1* and *kaku4* nuclei had small numbers of differential ATAC-seq peaks (*crwn1*, 36; *kaku4,* 46; Additional file 5: Table S4). Besides, our RNA-seq experiment showed that there were only 14 genes (including *CRWN1*) that became differentially expressed in *crwn1* seedlings (Additional file 6: Table S5). Thus, although *crwn1* nuclei showed different chromatin organization at a chromosomal level, they did not have massive alterations in chromatin accessibility or global transcriptome.

### *CRWN1* binds to non-accessible chromatin domains at the NP

**Fig. 6:**
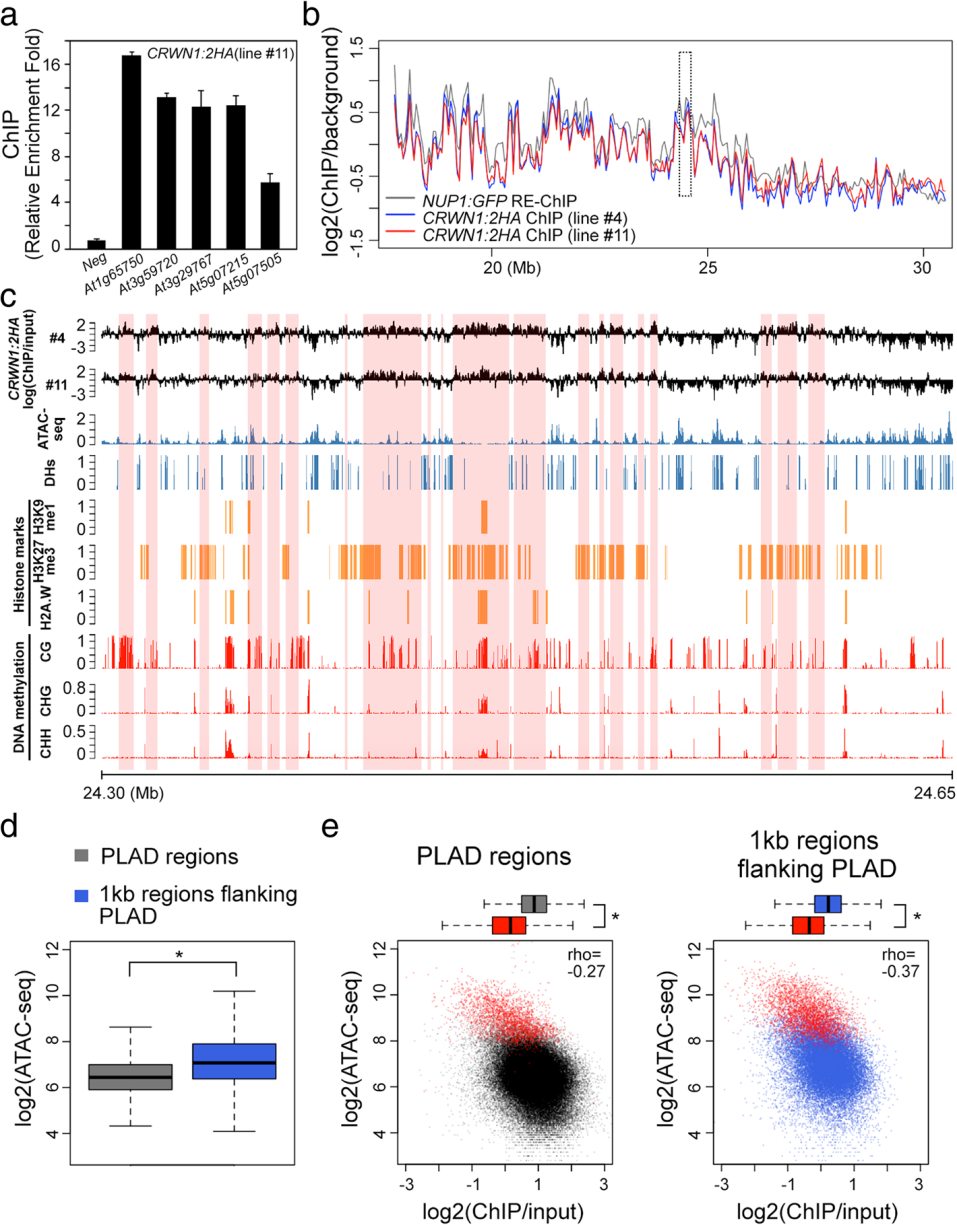
CRWN1 associates with non-accessible chromatin at the nuclear periphery. **a** *CRWN1:2HA* ChIP qPCR on selected loci that are located in chromatin region covered with Green probes shown in Fig. 1d. This panel shows a representative of three independent experiments. Error bars stand for standard deviation. **b** Plots of chromatin-NP association patterns, which are revealed by different approaches. For NUP1:GFP RE-ChIP, it is calculated as the logarithm of the ratio between anti-GFP and IgG [27]; for CRWN1:2HA ChIP, it is calculated as the logarithm of the ratio between anti-HA and input. This plot shows the right arm of chromosome 1 in 50-kb windows. A 350-kb region highlight with a dotted box is further shown in **c**, and this region overlaps with that covered by Green probes shown in Fig. 1. **c** Comparison of *CRWN1:2HA* ChIP signals with various structural and epigenomic features plotted in 100-bp windows. Shaded regions in pink depict PLADs. DHs, DNase I hypersensitive sites. **d** Comparison of ATAC-seq signals between PLADs and their 1-kb flanking regions. *p < 2.2 × 10^−16^ based on the Mann-Whitney *U* test. **e** Relationship between *CRWN1:2HA* ChIP and ATAC-seq signals in PLADs (left) and 1-kb flanking regions flanking PLADs (right). For each plot, chromatin regions overlapping with ATAC-seq peaks are colored in red; *p < 2.2 × 10^−16^ based on the Mann-Whitney *U* test

Given the functional relevance of CRWN1 in perinuclear chromatin positioning, we asked if there existed direct CRWN1-chromatin interactions. Firstly, we tested genomic loci in regions covered by Green probes shown in Fig. 1d, as their localization at the NP requires CRWN proteins. Chromatin immunoprecipitation experiments with a *CRWN1:2HA* tagging line, which fully rescued *crwn1* loss-of-function phenotypes, showed enrichment of CRWN1 at all candidate loci (Fig. 6a and Additional file 1: Figure S10). As expected, CRWN1:2HA proteins were primarily found at nuclear borders (Additional file 1: Figure S10d). Next, we performed ChIP-seq to study genome-wide patterns of CRWN1-chromatin contacts at the NP. Our data revealed that CRWN1 directly associated with chromatin regions with variable sizes (Additional file 7: Table S6). Remarkably, these regions, which we named plant lamina-associated domains (PLADs), largely overlapped with NP-enriched chromatin domains that we identified previously (Fig. 6b, Additional file 1: Figure S11 and Additional file 7: Table S6) [27]. These interstitial PLADs were enriched with repressive chromatin marks and lowly expressed genes, indicating that they were mainly transcriptionally inert regions (Additional file 1: Figures S12 and S13). By manually inspecting the ChIP-seq signal patterns, we found that PLADs did not have strong one-to-one relationships with heterochromatin marks, suggesting that the CRWN1-chromatin contacts were contributed by multiple factors (Fig. 6c). Interestingly, the CRWN1 ChIP-seq signals tended to be negatively correlated with marks indicative of accessible chromatin such as ATAC-seq peaks and DNaseI hypersensitive sites (Fig. 6c–e). Comparison of ATAC-seq signals between inside and outside PLADs revealed that the former had lower chromatin accessibility (Fig. 6d). Furthermore, for both chromatin regions inside and flanking PLADs, those overlapping with ATAC-seq peaks clearly showed lower CRWN1 ChIP-seq signals (Fig. 6e). Altogether, we conclude that via forming direct contacts with inaccessible chromatin, CRWN1 proteins mediate the docking of repressed chromatin to the nuclear lamina in plants.

## Discussion

The metazoan lamina plays crucial roles not only in regulating nuclear architecture by providing mechanical support, but also in modulating higher-order genome organization and a broad aspect of chromatin activities, which relies on selective chromatin tethering to the NP [1, 51]. With intensive efforts of identifying and characterizing components of the plant nuclear envelope, it is established that plants likely use a set of plant-specific proteins to build the lamina [52]. CRWNs, KAKU4, and NEAPs have been proposed as plant lamina constituents based on their protein domain structures, sub-nuclear localization patterns, and the nuclear architecture phenotypes of their loss-of-function mutants [12, 13, 17, 18]. However, it remains elusive whether or not they participate in tethering specific genomic regions. It is also unclear whether they are involved in regulating chromatin activities at the NP. In this study, we show that *Arabidopsis* lamin candidate proteins CRWN1 and CRWN4 possess abilities in mediating specific chromatin positioning at the NP—an important function that metazoan lamins have [34]. Earlier studies showing the involvement of CRWNs in regulating chromatin organization focused on highly heterochromatic chromocenters [14, 15]. Here, we generalize it to a chromosomal scale by showing that CRWN1 and CRWN4 are required to tether specific genomic regions on chromosome arms to the NP (Fig. 4 and Table 1). Remarkably, like metazoan lamin proteins, CRWN1 directly interacts with repressive chromatin domains positioned at the NP (Fig. 6). In addition, CRWN proteins positively regulate chromatin compartmentalization (Fig. 5), resembling the function of metazoan lamins in modulating the global three-dimensional genome interactions that has been reported recently [53, 54]. Taken together, we conclude that despite having no sequence homology with metazoan lamins, CRWN1 is functionally equivalent to them. CRWN proteins are found in all plant species, and the majority of characterized CRWNs show preferential localization at the NP [8, 10, 12, 13, 55]. We hypothesize that CRWNs have evolved across the plant kingdom to play central roles in mediating interactions between chromatin (likely heterochromatin) and the nuclear lamina.

It would be interesting to further elucidate how CRWN1 and CRWN4 would interact to regulate perinuclear chromatin anchoring in *Arabidopsis*. The relationship between CRWN1 and CRWN4 appears puzzling. At a physiological level, they clearly show functional redundancy because of the growth retardation phenotype developed in *crwn1 crwn4* double mutants [14]. However, at a molecular level, one may conclude that these genes have partial antagonizing roles. According to a recent transcriptomic study of *crwn* mutants, the number of up-/downregulated genes in *crwn1*, *crwn4*, *and crwn1 crwn4* plants was 455/271, 1539/1151, and 395/329, respectively [56]. Thus, the loss of function of *CRWN1* can largely suppress global changes in gene expression in the *crwn4* background. To unveil the potential redundant, cooperative, or antagonistic nature of these two CRWNs in regulating perinuclear chromatin positioning, one can perform a series of experiments to compare how chromatin-NP interactions differ from one another among single and higher-order *crwn* mutants.

The molecular mechanisms by which LADs are brought to the NP in animals are not fully understood [1]. During *C*. *elegans* embryogenesis, this relies on the repressive mark, H3K9me [39, 57, 58]. However, in differentiated *C*. *elegans* cells, the anchoring of LAD at the NP is partially retained in the absence of H3K9me, suggesting the existence of different mechanisms regulating perinuclear chromatin localization at different developmental stages [39]. Our findings suggest that chromatin-NP interactions in seedlings are not affected when plants lose the H3K9me mark (Fig. 3 and Table 2). In agreement with this notion, pericentromeric regions had been shown to be maintained at the NP in *suvh4 suvh5 suvh6* triple mutants that lost H3K9me [59]. Certainly, we could not exclude a possibility that H3K9me is essential for chromatin-NP interactions in other plant tissues.

As PLADs are enriched with transposons and lowly expressed protein-coding genes, one can expect seeing enrichment of any given heterochromatic mark in PLADs comparing to non-PLAD regions (e.g., H3K27me1, H2A.W, H3K27me3, and DNA methylation shown in Additional file 1: Figure S12). Thus, carrying out comparative FISH analysis in mutant plant nuclei losing each of these marks would be necessary to verify whether or not they are required for perinuclear chromatin anchoring. Interestingly, we found that chromatin-NP interactions in *Arabidopsis* could be manipulated by combining mutants of CHG and CHH methylation pathways (Fig. 3 and Table 2). Mutation in the RdDM pathway alone, which is part of the CHH DNA methylation mechanisms, seems not enough to disrupt chromatin-NP interactions, since the loss-of-function mutations in *DRM1* and *DRM2* did not abolish specific perinuclear chromatin localization (Fig. 3 and Table 2). Consistently, other mutants deficient of the RdDM pathway (e.g., *nrpd1* and *nrpe1*) showed similar results (Additional file 1: Figure S14). We noticed that although transposable elements covered only 7% of PLADs, they showed stronger interactions with CRWN1 than did other types of PLAD regions (Additional file 1: Figure S13c). Based on this observation, we propose a following mechanism that contributes to *Arabidopsis* perinuclear chromatin distribution patterns: the non-CG DNA methylation in the genome can be read by certain factor(s) yet to be determined (perhaps a protein complex containing CRWNs), which gives rise to the initial contacts between chromatin and the nuclear envelope. Subsequently, the pre-formed chromatin-NP contacts further spread to include additional inaccessible genomic loci adjacent to them.

It is interesting that alterations of the three-dimensional chromatin organization and the loss of specific chromatin-NP interactions in *crwn1* did not cause widespread changes in local chromatin accessibility or gene expression (Additional file 5: Table S4 and Additional file 6: Table S5). Evaluating the effects of global loss of chromatin-NP interactions in different metazoan species suggests that they may contribute to a varying degree to gene repression, depending on species and cell type [1]. In our view, caution should be paid in assessing how important CRWN1-mediated chromatin-NP interactions are to transcriptional regulation, as CRWN2 and CRWN3, which are homologs of CRWN1, are localized throughout the nucleoplasm [12, 13, 60]. These CRWNs might act as backups to maintain chromatin structure and gene expression when a chromatin region originally tethered at the NP detaches from it. According to phenotypic and expression analyses of single and higher-order *crwn* mutants, it is clear that CRWN proteins share functional redundancy in regulating heterochromatin organization, salicylic acid synthesis, and the protection of genomic DNA against excessive oxidation [14, 22, 56, 61]. However, it is challenging to clarify if these phenotypes are linked to transcriptional regulation imposed directly by CRWNs or to severe changes in nuclear morphology in the higher-order *crwn* plants [14]. Instead, switching to other model plant species with less CRWN genes might be helpful to address this question.

Although *Arabidopsis* CRWN1 or other CRWNs seem to have stable transcriptional activities under biotic and abiotic stress conditions (expression data integrated by [62]), they might undergo intensive post-translational modifications (PTMs). A recent study revealed that upon *Pseudomonas syringae pathovar maculicola* infection, CRWN1 proteins were quickly degraded, which was mediated by the salicylic acid (SA) signaling pathway [22]. Given the role of CRWN1 in regulating chromatin positioning and chromosome territory, one can hypothesize that when the SA signaling pathway becomes highly activated under stress, the downregulation of CRWN1 protein will give rise to an alternative chromatin organization as part of stress responses. Metazoan lamins are subject to phosphorylation at various sites, which influence how they interact with themselves and other molecules (reviewed recently by [63]). For instance, different phosphorylation patterns of lamins can regulate how they polymerize and affect the stiffness/plasticity of the lamina network, how they are distributed in the nuclear compartments (i.e., nucleoplasm-associated vs. lamina-associated), and how they interact with transcription factors, chromatin remodeling factors, and chromatin [63, 64]. It is worthy to note that according to a plant protein phosphorylation database (http://dbppt.biocuckoo.org) [65], CRWN1 has over 30 identified phosphorylation sites, most of which are located outside its coiled-coil rod domain, and the total number for phosphorylation sites in CRWN1 is way more than that of any other *Arabidopsis* CRWNs. This information implies that phosphorylation might be an essential mark regulating CRWN1. Apart from phosphorylation, many other types of PTMs of metazoan lamins, such as sumoylation, acetylation, glycosylation, and farnesylation, have been documented [66]. These PTMs, however, have not been described in plants. In the future, characterizing various PTMs of plant lamins and resolving their potential crosstalk would be inspiring, probably critical, steps to understanding how lamin proteins regulate plant growth and development.

## Conclusions

We reveal that perinuclear chromatin anchoring in the model plant species *Arabidopsis thaliana* requires CRWN1, CRWN4, and non-CG DNA methylation, which are all plant-specific. We show that CRWN1 has direct interactions with inaccessible chromatin at the nuclear periphery, and it is involved in maintaining chromatin compartmentalization, suggesting that it is functionally equivalent to animal lamin proteins. As CRWNs are highly conserved across plants, our work provides a platform for further exploring and investigating the interplay between plant chromatin and the nuclear envelope.

## Methods

### Plant material and nucleus sorting

All *Arabidopsis* plants used in this study were grown at 23 °C in long days (16 h light/8 h dark) on half-strength Murashige and Skoog (MS) medium supplemented with 1% sucrose and 0.3% Phytagel. Mutants used in this study were *crwn1-1* (SALK_025347), *crwn4-1* (SALK_079296), *kaku4-2* (SALK_076754), *morc6-3* (GABI_599B06), *neap1-1* (SAIL_846_B07), *neap3-1* (GABI_221C05), *nrpd1-3* (SALK_128428), *nrpe1-11* (SALK_029919), *suvh4 suvh5 suvh6* (SALK_041474, GABI_263C05, SAIL_1244_F04), *cmt2-3* (SALK_012874), *cmt3-11* (SALK_148381), *drm1-2 drm2-2* (SALK_031705, SALK_150863), *met1-3* [67], and their derived higher-order mutants. All mutant lines used in this study were in Columbia background. For collecting *met1-3* nuclei, only the first generation of homozygous plants derived from *met1-3/MET1* heterozygous parents was used.

Nuclei were collected from the aerial parts of 10-day-old seedlings. Nucleus extraction and sorting were performed essentially according to steps described in our earlier work [45]. The extracted nuclei were stained with 0.5 μM DAPI to reveal their ploidy levels; only 2C nuclei were collected for downstream experiments. Note that our 2C nuclei pool was consisting of a mixture of different types of cell nuclei, because there was no tissue-specific marker used for further selection. Nonetheless, we considered that the majority was from mesophyll cells.

### Plasmid construction

*CRWN1:2HA* was constructed via performing overlapping PCR of two fragments, with which a tandem HA tag was inserted into CRWN1. For one fragment, the genomic fragment spanning 1.7 kb upstream of *CRWN1*’s transcription start site to the corresponding nucleotide for the 780th amino acid residue of CRWN1 was amplified with primers 5′-TTACGTTTTATTGTGGTCTTC-3′ and 5′-AGGGTATCCAGCATAATCTGGTACGTCGTATGGGTATCCAGCAGTTGGGGATATATCCC-3′. The other fragment consisting of the rest coding region of *CRWN1* plus 0.7 kb downstream was amplified with primers 5′-GATTATGCTGGATACCCTTACGACGTACCAGATTACGCTGCTGGCTTAGGATTGCCAGTT-3′ and 5′-ATAATACTGTCAAGAGTGATG-3′. These two fragments were assembled with overlapping PCR and amplified with primers 5′-TTACGTTTTATTGTGGTCTTC-3′ and 5′-ATAATACTGTCAAGAGTGATG-3′. The final PCR product was cloned into a Gateway-compatible pGREEN-IIS binary destination vector [68].

### FISH and immunohistostaining

BACs selected for FISH were labeled with digoxigenin-11-dUTP or dinitrophenol-11-dUTP by nick translation, which were detected with Alexa Fluor 488 (Green) or Alexa Fluor 546 (Red) according to our dual-color FISH protocol, respectively [27]. We collected around 5000 sorted 2C nuclei for one hybridization spot (~ 1 cm^2^). After nucleus sorting, the nuclei were centrifuged for 1000*g* at 4 °C for 5 min, and the pellet was resuspended with 10 μl PBS buffer. The nuclei were incubated at 65 °C for 15 min and mixed with 5 μl 0.1 mg/ml RNase A. The mixture were transferred onto a Superfrost Ultra Plus Adhesion Slide (ThermoFisher Scientific) and incubated for 1 h at 37 °C. At the end of RNase A treatment, the nuclei became attached to the glass slide. Next, the slide was washed briefly with PBS buffer and dehydrated in a graded series of alcohol solutions. All subsequent steps, including probe denaturation, hybridization, washing, and detection, were performed according to [27] with minor changes. For probes targeting regions shown in Fig. 1d, the concentration of each labeled BAC was adjusted to 1 ng/μl in the working hybridization solution. For probes used for chromosome painting shown in Fig. 4, the concentration of each labeled BAC in the working hybridization solution was 0.05 ng/μl. Information of BACs used in this study is provided in Additional file 2: Table S1.

For immunohistostaining, *CRWN1:2HA crwn1* seedlings were fixed and embedded in paraffin as described [69]. Slides with paraffin sections of leaf tissues were dewaxed and rehydrated with PBS buffer. After that, an antigen retrieval step was performed with Universal HIER antigen retrieval reagent (abcam) by following the manufacturer’s instructions. The slides were incubated with 1:500 diluted HA Tag Alexa Fluor 647 conjugate (ThermoFisher Scientific). After washing, slides were mounted with SlowFade® Diamond Antifade Mountant with DAPI (ThermoFisher Scientific).

### Fluorescence microscopy and image processing

Confocal images were acquired with a Zeiss LSM 880 Airyscan system.

For measuring the distance of short genomic intervals to the NP (shown in Fig. 1d, Table 1, and Table 2), a single image was taken for a nucleus if it had at least one distinct green and red signal spots at the same focal plane. The distance of a signal spot to the NP was recorded as the distance between its estimated barycenter and the edge of DAPI staining. See Additional file 1: Figure S15 for an example.

Chromosome painting data (shown in Fig. 4) were acquired as z-stack. For each image, we used ImageJ [70] to determine nuclear boundary, while signals in green and red channels were extracted from the corresponding image files (Additional file 1: Figure S2). We found that the nuclear part close to the glass slide tended to become flattened, probably due to capillary forces. To reduce possible errors in distance calculation in this area, the bottom two images of each nucleus were not included so that our data analyses were only applied to a subset of the nuclear space. As nuclei landed on slide randomly, FISH signal distribution along the *z*-axis is independent from distance to the nuclear boundary. Such data exclusion, in principle, should not affect our conclusion concerning signal distribution in the remaining part of the nucleus.

### In situ Hi-C

The in situ Hi-C library preparation was performed mainly by following a protocol optimized for *Arabidopsis* seedlings [45]. Because the amount of input nuclei used for Hi-C experiment in this study was much less than that of a regular scale, the following modifications were made.

Hi-C was performed with two biological replicates. For each replicate, 200,000 2C nuclei were collected in PBS buffer and centrifuged 3000*g* at 4 °C for 5 min. The pellet was resuspended with 12.5 μl 0.5% SDS and incubated at 62 °C for 5 min. The excessive SDS were quenched by adding 36.25 μl water and 6.25 μl 10% Triton X-100 and incubated at 37 °C for 15 min. Subsequently, chromatin was digested by adding 6.25 μl 10× DpnII buffer (10 mM MgCl_2_, 1 mM DTT, 100 mM NaCl, 50 mM Bis-Tris-HCl, pH 6.0), 10 U of DpnII, and incubated at 37 °C overnight. Next, the DpnII enzyme was inactivated at 62 °C for 15 min. To fill the cohesive ends generated by DpnII, the nuclei were incubated with 0.25 μl 10 mM dTTP, 0.25 μl 10 mM dATP, 0.25 μl 10 mM dGTP, 6.25 μl 0.4 mM biotin-14-dCTP, 3.5 μl water, and 1 μl (10 U) Klenow, and incubated at 37 °C for 2 h. Next, ligation of chromatin was performed by adding 166 μl water, 30 μl 10× blunt-end ligation buffer (300 mM Tris-HCl, 100 mM MgCl_2_, 100 mM DTT, 1 mM ATP, pH 7.8), 25 μl 10% Triton X-100, and 5 U T4 DNA ligase, and the mixture was incubated at room temperature for 4 h. After ligation, nuclei were collected by centrifugation at 1000*g* for 3 min. The pellet was resuspended with 150 μl SDS buffer (50 mM Tris-HCl, 1% SDS, 10 mM EDTA, pH 8.0) and incubated with 10 μg proteinase K at 55 °C for 30 min. After that, 8 μl 5 M NaCl was added, and the sample was incubated at 65 °C overnight for decrosslinking. On the next day, the Hi-C DNA was purified according to [47].

The purified Hi-C DNA was sheared to fragments shorter than 500 bp with a Covaris E220 sonicator by using following settings: duty factor, 10; peak incident power, 170; cycles per burst, 200; and treatment time, 60 s. The sonicated DNA was purified by using Ampure beads with a size cutoff for selective recovering DNA longer than 300 bp. Next, with a 50 μl reaction volume, the DNA was mixed with 0.5 μl 10 mM dTTP, 0.5 μl 10 mM dATP, and 5 U T4 DNA polymerase and incubated at 20 °C for 30 min to remove biotin from unligated ends. After that, the DNA was purified with Ampure beads. Subsequently, DNA end-repair and adaptor ligation were performed with the NEBNext® Ultra™ II DNA Library Prep Kit by following the manufacturer’s instructions. The ligated DNA was further affinity-purified with Dynabeads MyOne Streptavidin C1 beads (Invitrogen) as described by [47], Finally, library molecules were amplified with 16 PCR cycles and sequenced on an Illumina HiSeq 3000 instrument with 2 × 150 bp reads.

### Hi-C read mapping, filtering, and Hi-C map normalization

Read mapping, removal of PCR duplicates, and read filtering were performed as described [29]. Hi-C reads from each biological replicate of wild-type and *crwn* mutants are summarized in Additional file 3: Table S2. Generation of normalized Hi-C maps was done according to [45] with an iterative matrix correction function in the “HiTC” package in R [71]. The two replicates were merged and used to generate Hi-C maps, except for Additional file 1: Figure S3 that reads of each replicate were processed separately. For all Hi-C maps, the iterative normalization process was stopped when the eps value, which reflected how similar the matrices in two consecutive correction steps were, dropped below 1 × 10^−4^. Normalization at 20-kb resolution was done at a genome-wide level (i.e., all chromosomes were included), while normalization at 10-kb resolution was done for each chromosome separately.

### ATAC-seq

ATAC-seq was performed with two biological replicates. For each replicate, 20,000 of sorted 2C nuclei were collected in PBS buffer and centrifuged at 3000*g* at 4 °C for 5 min. The nuclei were resuspended with 20 μl Tn5 transposase (Illumina) reaction prepared according to [60]. The transposed DNA was purified with MinElute PCR Purification Kit (Qiagen) and amplified with selected Nextera index oligos (Illumina). Size selection of PCR products was performed with AMPure® XP beads (Beckman Coulter) to collect library molecules between 200 and 600 bp. Finally, the purified libraries were pooled and sequenced.

### ATAC-seq reads processing and peak calling

ATAC-seq reads were aligned against the *Arabidopsis thaliana* reference genome (TAIR10) using Bowtie 2 v2.2.4 [72] with a “very sensitive” mapping mode. Duplicate reads were removed during the ATAC-seq peak calling process with MACS2 or during the generation of sequencing coverage files with Bedtools (see below). ATAC-seq peaks (i.e., accessible chromatin regions) in each genotype were identified with MACS2 [73] using settings as “nomodel --shift -50 --extsize 100 --keep-dup=1”, and peaks with false discovery rate smaller than 0.05 were retained. Subsequently, ATAC-seq peaks identified in 2C nuclei of each genotype (wild-type, *crwn1*, and *kaku4*) were merged, which gave rise to a master list of potentially accessible regions. Calling differential ATAC-seq peaks was performed according to [74]. Briefly, a count table describing the sequencing coverage of these accessible regions in each sample was generated with the *bedtools multicov* command, using the sorted bam mapping files as input. This count table was used to identify differential peaks with the DESeq2 package in R [75]. We used the criteria of false discovery rate smaller than 0.05 and fold change greater than 1.5 to call gain-of-accessibility (UP) and loss-of-accessibility (DOWN) peaks. The ATAC-seq peaks called from each genotype, the peak count table, and the differential peaks can be found in Additional file 5: Table S4.

### RNA-seq library preparation and analysis

RNA-seq was performed with three biological replicates. The total RNA was isolated from 10-day-old aerial parts of seedlings with RNeasy Plant Mini Kit (Qiagen), and libraries were prepared according to a standard protocol from Illumina with minor modifications. Briefly, 500 ng of DNase I (Thermo Scientific, Waltham, MA, USA) treated RNA was used for library preparation. The RNA molecules were mixed with First Strand buffer (Invitrogen), heat fragmented at 80 °C for 2 min followed with 94 °C for 1.5 min. Next, the fragmented RNA molecules were used for synthesizing the first and second strand of cDNA. After that, cDNA molecules were repaired, A-tailed, and ligated with barcoded adaptors. The ligated samples were then enriched by amplification and purified for sequencing. RNA-seq reads alignment and further downstream processing were performed according to [45]. We used the criteria of false discovery rate smaller than 0.05 and fold change of greater than 2 to call differentially expressed genes.

### ChIP, ChIP-seq, and data analysis

Tissue fixation, nucleus isolation, and chromatin fragmentation were performed according to [29]. After sonication, chromatin was immunoprecipitated with 12 μl of Pierce™ Anti-HA Magnetic Beads (ThermoFisher Scientific) at 4 °C for 2 h. The ChIP-ed DNA was extracted with MinElute PCR Purification Kit (Qiagen). The purified DNA was either used for qPCR or converted into sequencing libraries with NEBNext® Ultra™ II DNA Library Prep Kit (NEB). Libraries were sequenced on an Illumina HiSeq 3000 instrument with 2 × 150 bp reads. Calculation of fold enrichment with quantitative real-time PCR was performed as described previously [27]. The relative abundance of each tested locus was normalized to *TUB2*. Oligos used for ChIP-qPCR are listed in Additional file 8: Table S7.

For ChIP-seq analysis, reads were aligned against *Arabidopsis thaliana* reference genome (TAIR10) using Bowtie 2 v2.2.4 [72] with a “very sensitive” mapping mode. The mapped reads were analyzed by SICER v1.1 [76] to call enriched regions (parameters: *W* = 500; *G* = 1500; FDR < 0.01). The enriched chromatin regions obtained from two independent transgenic lines (#4 and #11) were compared, and those shared between the two replicates were annotated as PLADs.

## Additional files

Additional file 1: **Figure S1.** Nuclear morphology of lamin-like gene mutants. **Figure S2.** Approximation of chromosome painting data. **Figure S3.** Comparison of Hi-C maps. **Figure S4.** Overview of Hi-C maps of wild-type and *crwn* mutants.** Figure S5.** *crwn* mutants show more inter-compartment chromatin contacts. **Figure S6.** Inter-chromosomal interactions in wild-type and *crwn* plants. **Figure S7.** Inter-chromosomal interactions of chromatin regions tethered at the nuclear periphery. **Figure S8.** ATAC-seq as a tool to reveal differential chromatin organization patterns and changes in chromatin packing. **Figure S9.** Chromatin accessibility is not affected in *crwn1* or *kaku4* mutants. **Figure S10.** A native *CRWN1* tagging construct can fully rescue *crwn1* phenotypes. **Figure S11.** Comparison of chromatin-NP interaction patterns revealed from different methods. **Figure S12.** Features associated with PLADs and their flanking chromatin regions. **Figure S13.** Genes associated with PLADs. **Figure S14.** Analyses of FISH signals in RdDM mutants. **Figure S15.** Representative confocal images showing the localization of probes in WT and *crwn1* 2C nuclei. (DOCX 8994 kb)

Additional file 2: **Table S1.** BAC vectors used for making FISH probes. (XLSX 15 kb)

Additional file 3: **Table S2.** Statistics of Hi-C reads. (XLSX 9 kb)

Additional file 4: **Table S3.** Differential accessible chromatin regions in *morc6* 2C nuclei. (XLSX 80 kb)

Additional file 5: **Table S4.** ATAC-seq count table and peaks among wild-type, *crwn1*, and *kaku4*. (XLSX 12773 kb)

Additional file 6: **Table S5.** RNA-seq count table and differentially expressed genes. (XLSX 3200 kb)

Additional file 7: **Table S6.** Plant lamina-associated domains. (XLSX 64 kb)

Additional file 8: **Table S7.** Oligos used for ChIP-qPCR. (XLSX 18 kb)
