# Supplementary material for: Plant lamin-like proteins mediate chromatin tethering at the nuclear periphery
Source: Genome Biol. 2019 Apr 30;20:87. doi: 10.1186/s13059-019-1694-3 (PMC6492433; doi:10.1186/s13059-019-1694-3)
Supplement: Supplementary file 1 — Figure S1. Nuclear morphology of lamin-like gene mutants. Figure S2. Approximation of chromosome painting data. Figure S3. Comparison of Hi-C maps. Figure S4. Overview of Hi-C maps of wild-type and crwn mutants. Figure S5. crwn mutants show more inter-compartment chromatin contacts. Figure S6. Inter-chromosomal interactions in wild-type and crwn plants. Figure S7. Inter-chromosomal interactions of chromatin regions tethered at the nuclear periphery. Figure S8. ATAC-seq as a tool to reveal differential chromatin organization patterns and changes in chromatin packing. Figure S9. Chromatin accessibility is not affected in crwn1 or kaku4 mutants. Figure S10. A native CRWN1 tagging construct can fully rescue crwn1 phenotypes. Figure S11. Comparison of chromatin-NP interaction patterns revealed from different methods. Figure S12. Features associated with PLADs and their flanking chromatin regions. Figure S13. Genes associated with PLADs. Figure S14. Analyses of FISH signals in RdDM mutants. Figure S15. Representative confocal images showing the localization of probes in WT and crwn1 2C nuclei. (DOCX 8994 kb) [file 13059_2019_1694_MOESM1_ESM.docx]

**Plant lamin-like proteins mediate chromatin tethering at the nuclear periphery**

Bo Hu, Nan Wang, Xiuli Bi, Ezgi Süheyla Karaaslan, Anna-Lena Weber, Wangsheng Zhu, Kenneth Wayne Berendzen, and Chang Liu


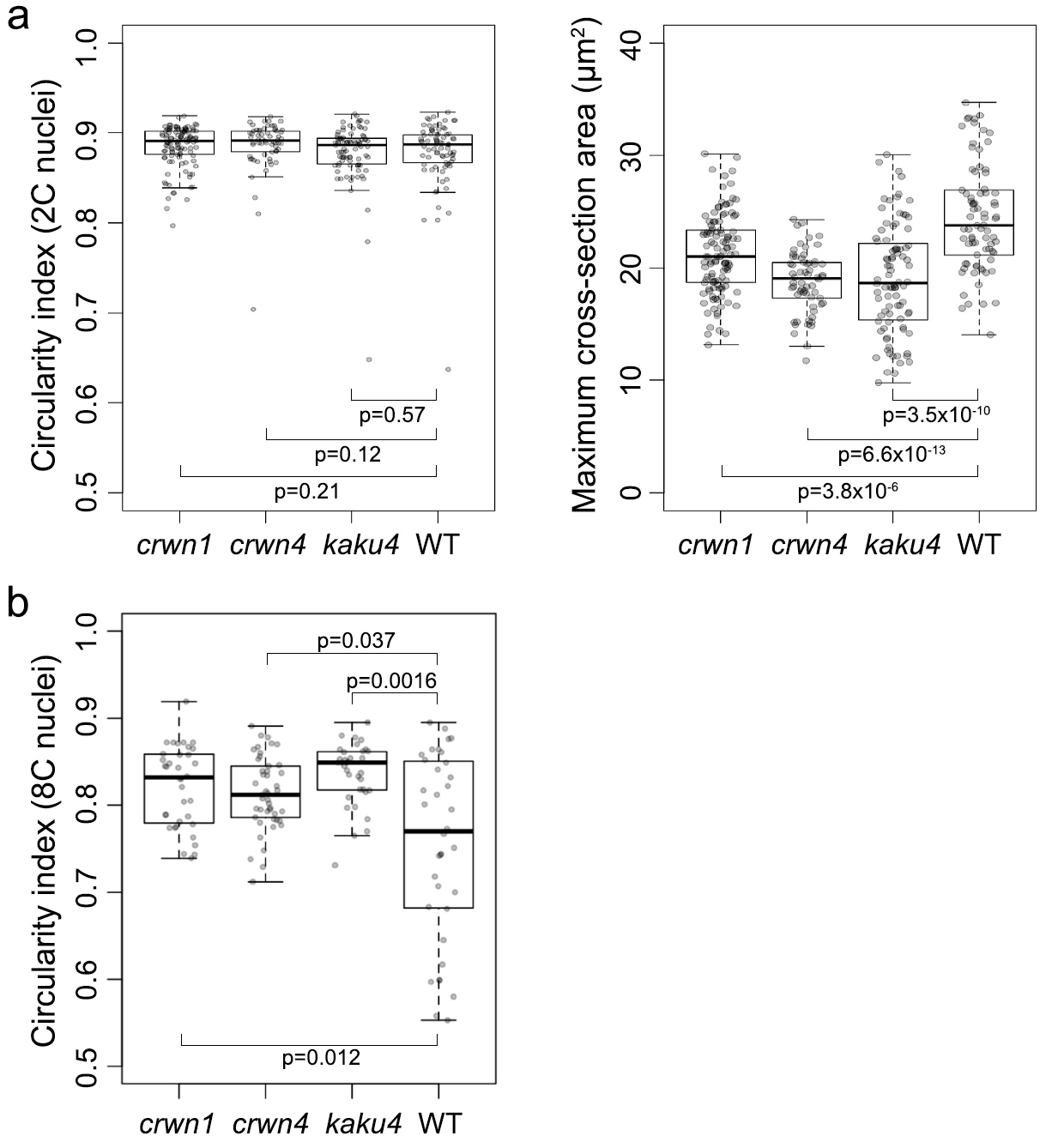


**Figure S1. Nuclear morphology of lamin-like gene mutants.**

**a,b.** 2C nuclei **(a)** and 8C nuclei **(b)** from various plant seedlings were compared. The circularity index was calculated as 4πA/P^2^, where A and P were the area and perimeter of a nucleus retrieved from confocal images, respectively. The large variation of the circularity index in the wild-type sample in **(b)** is due to the presence of different types of endoreduplicated nuclei. Generally, mesophyll cell nuclei are more spherical than those of pavement and vasculature cells. *p*-values indicate Mann-Whitney U test results. WT, wild-type.


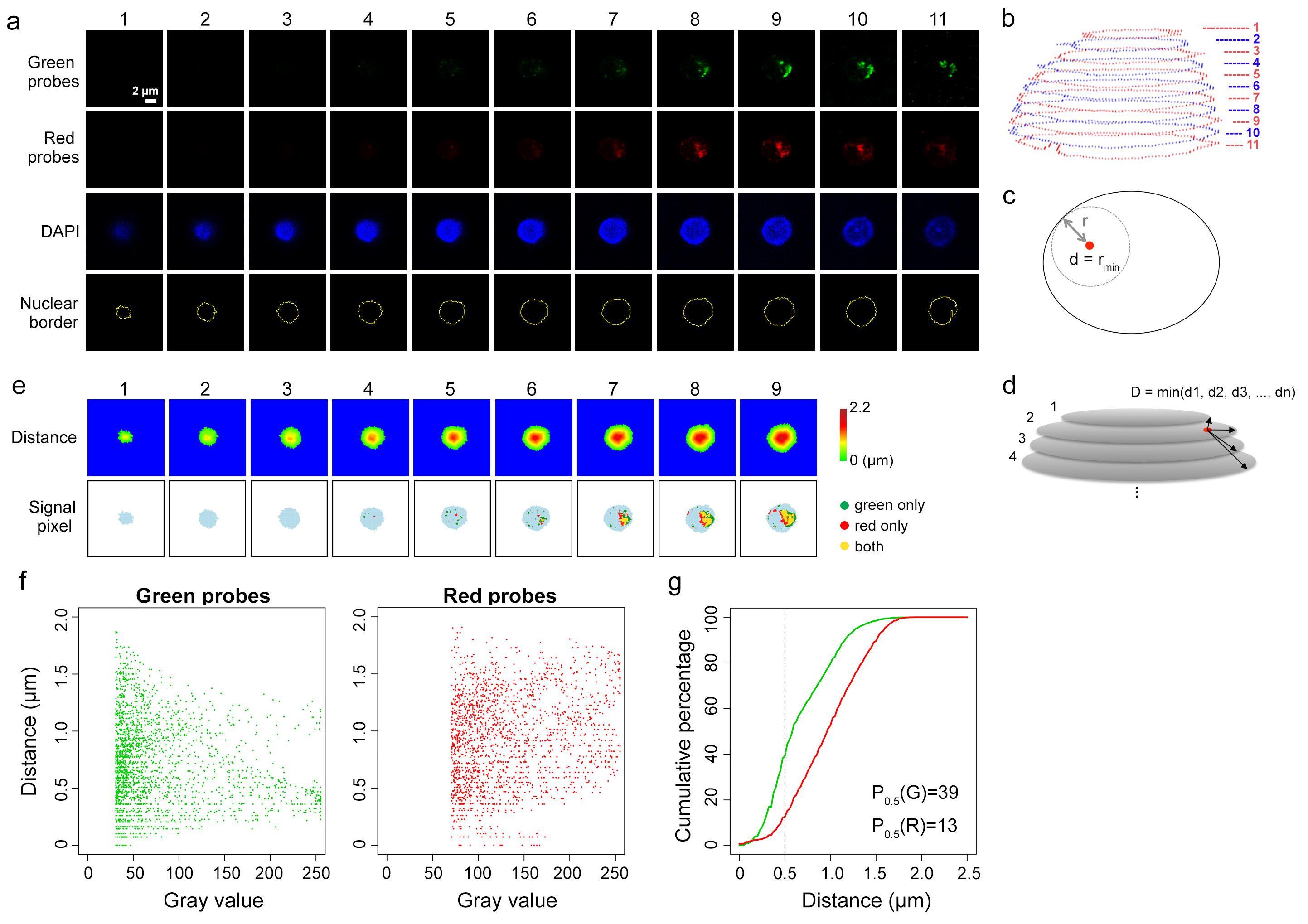


**Figure S2. Approximation of chromosome painting data.**

**a.** Z-stack confocal image files of a wild-type 2C nucleus (its z-projection image is shown in Fig. 4b). The bottom row indicates nuclear border of each slice identified from the corresponding DAPI image.

**b.** Reconstructed nucleus. The bottom two slices (slices no. 10 and 11), which are close to the glass slide, are not included for further analyses.

**c,d.** Computing the distance to the nuclear periphery. **(c)** Within an optical slice, the distance of a given point (red) to the nuclear border is determined as the radius of the minimal circle intersecting with the nuclear border. **(d)** The distances of this point to the nuclear borders in other optical slices are calculated as well. At the end, the minimum value is defined as the distance of this point to the nuclear periphery.

**e.** Processed confocal images showing the distance of each pixel to the nuclear periphery (top row) and pixels with FISH signal (bottom row).

**f.** Relationship between FISH signal strength and distance to the nuclear periphery. **g.** Comparison of cumulative green and red FISH signals as a function of distance to the nuclear periphery. With a distance cutoff of 0.5 µm, for the analyzed optical slides in this nucleus, 39 % and 13 % of green and red signal is located at the nuclear periphery, respectively.


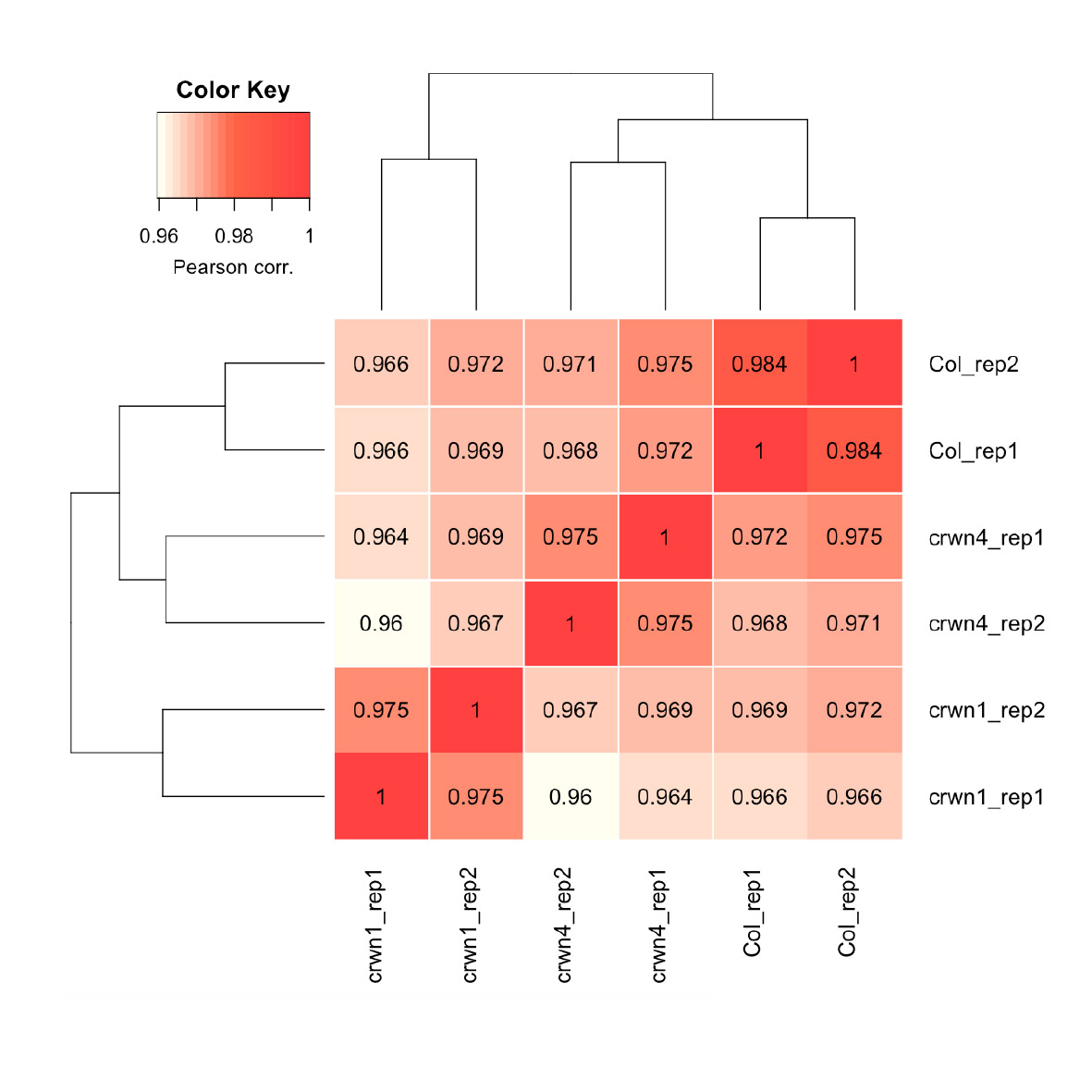


**Figure S3. Comparison of Hi-C maps.**

Genome-wide Hi-C maps (resolution: 20 kb) of various samples are compared. The dendrogram shows hierarchical clustering based on Euclidean distance.


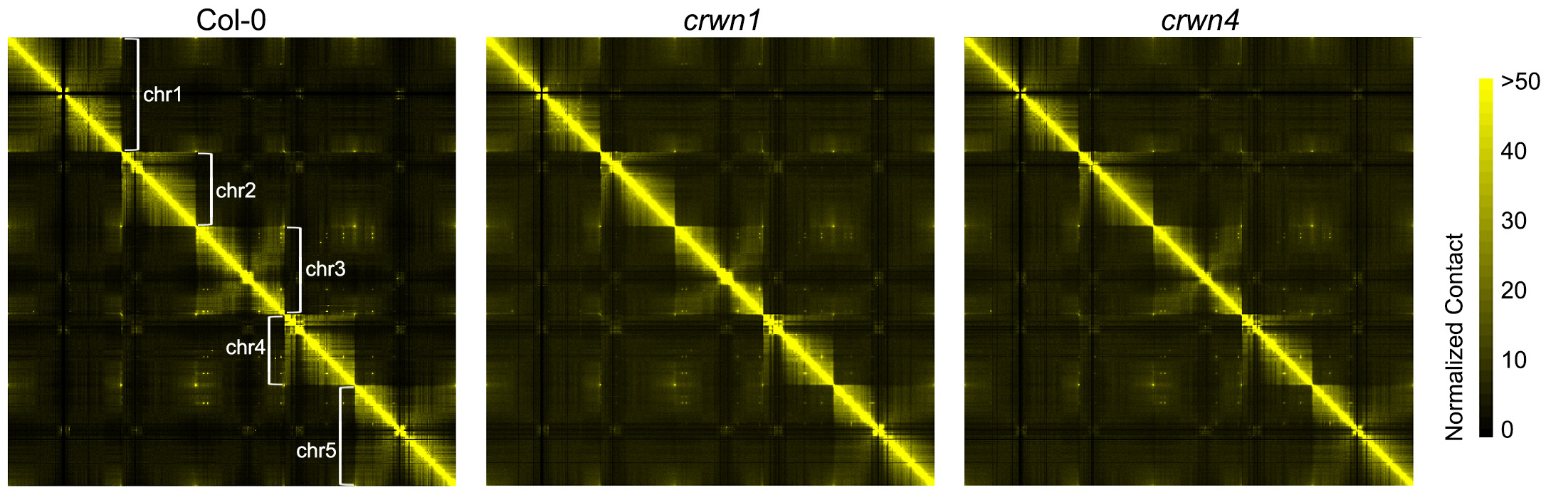


**Figure S4. Overview of Hi-C maps of wild-type and *crwn* mutants.**

Each Hi-C map is normalized with 20 kb bins.


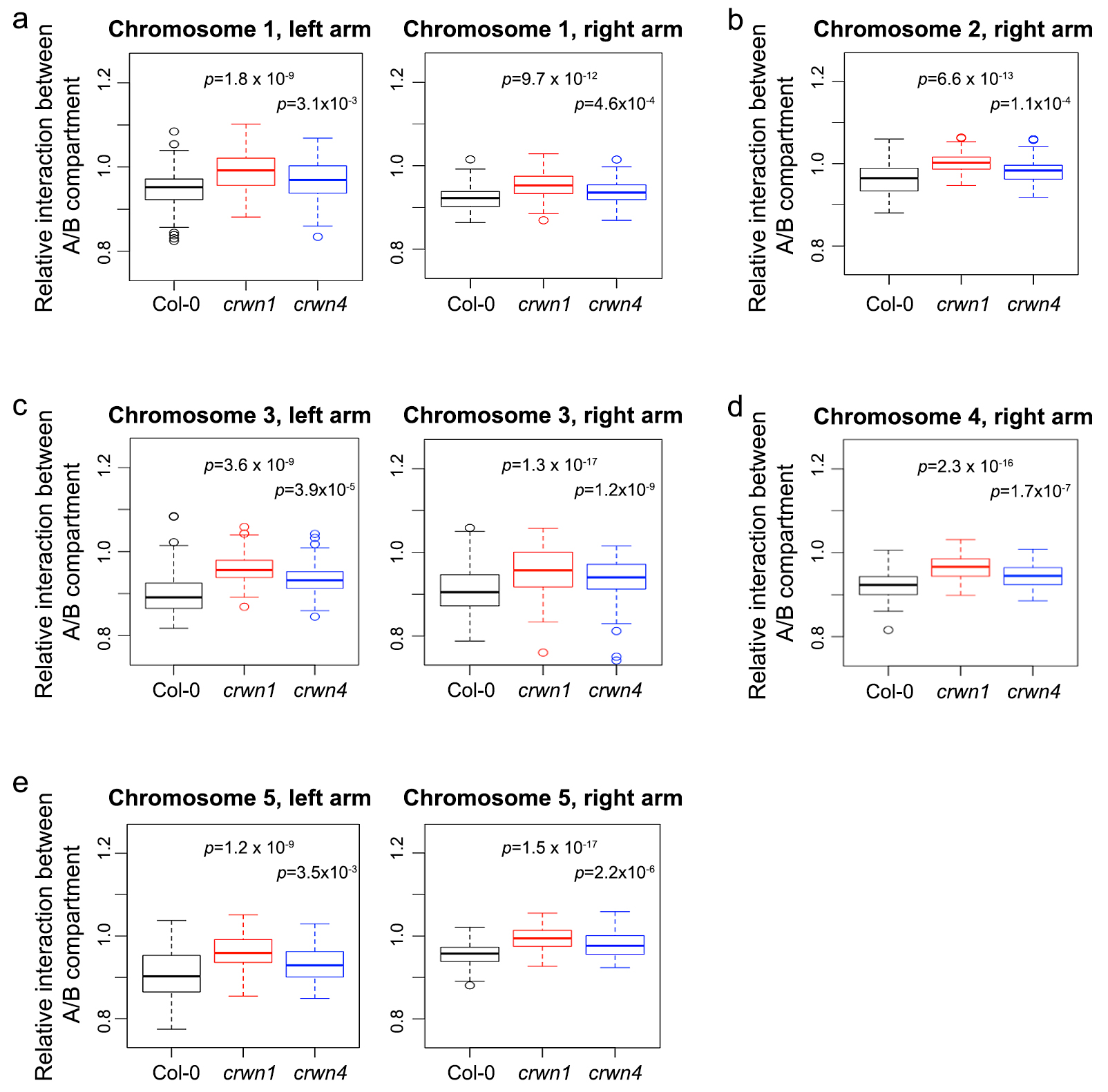


**Figure S5. *crwn* mutants show more inter-compartment chromatin contacts.**

**a-e.** Distance-normalized interaction strengths between A and B compartments relative to the average are calculated for each chromosome arm except for the short arm of chromosomes 2 and 4. *p* values indicate Mann-Whitney U test results. Results of chromosome 1 right arm are also shown in Fig. 5g.


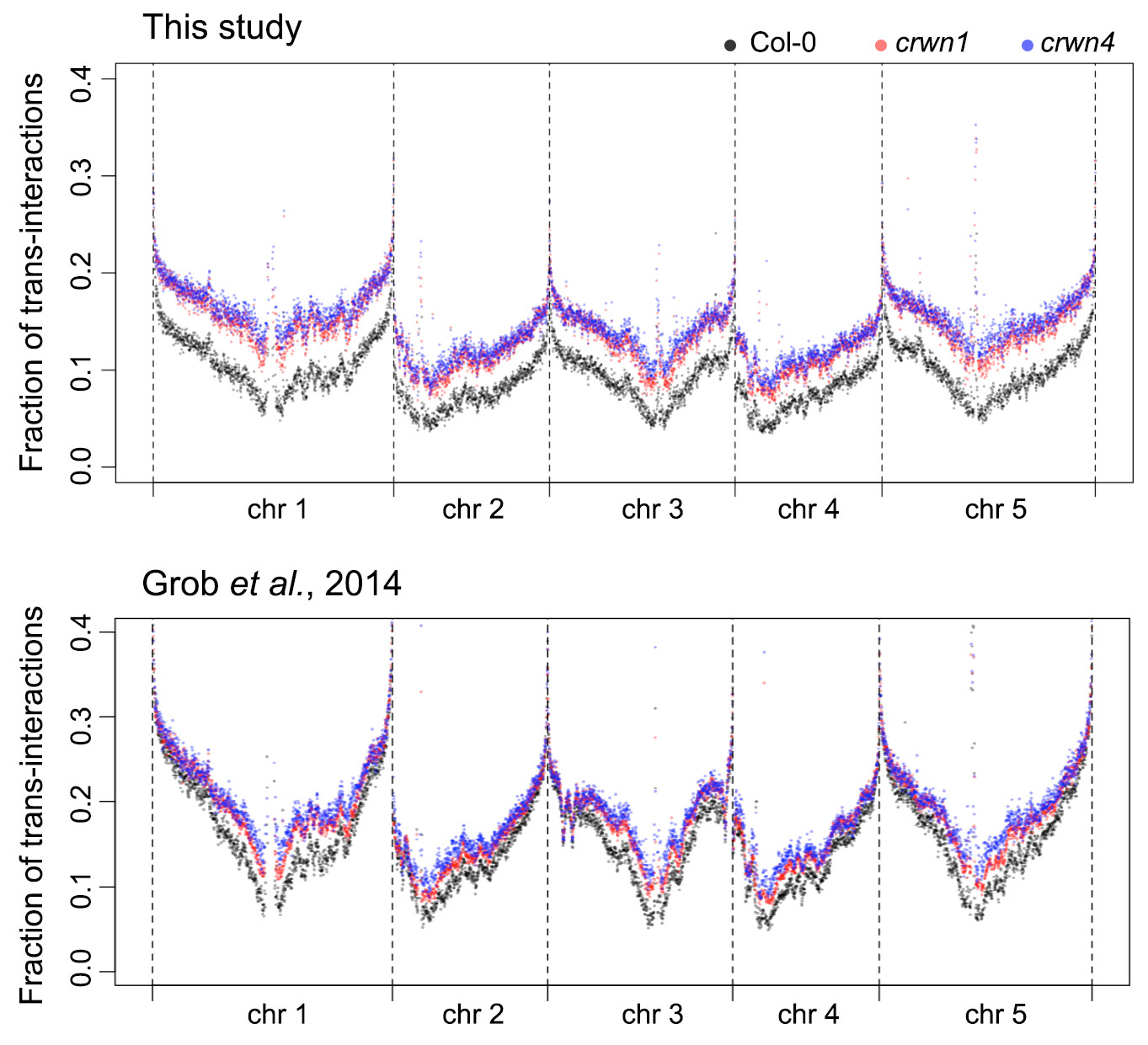


**Figure S6. Inter-chromosomal interactions in wild-type and *crwn* plants.**

For each genotype, the fraction of inter-chromosomal (trans-) interactions among the sum of intra- and inter-chromosomal interactions of each bin (20 kb in the upper panel; 25 kb in the lower panel) is calculated from the corresponding genome-wide Hi-C map. The Hi-C maps of Grob et al. were from normalized with 25 kb bins [1].

**
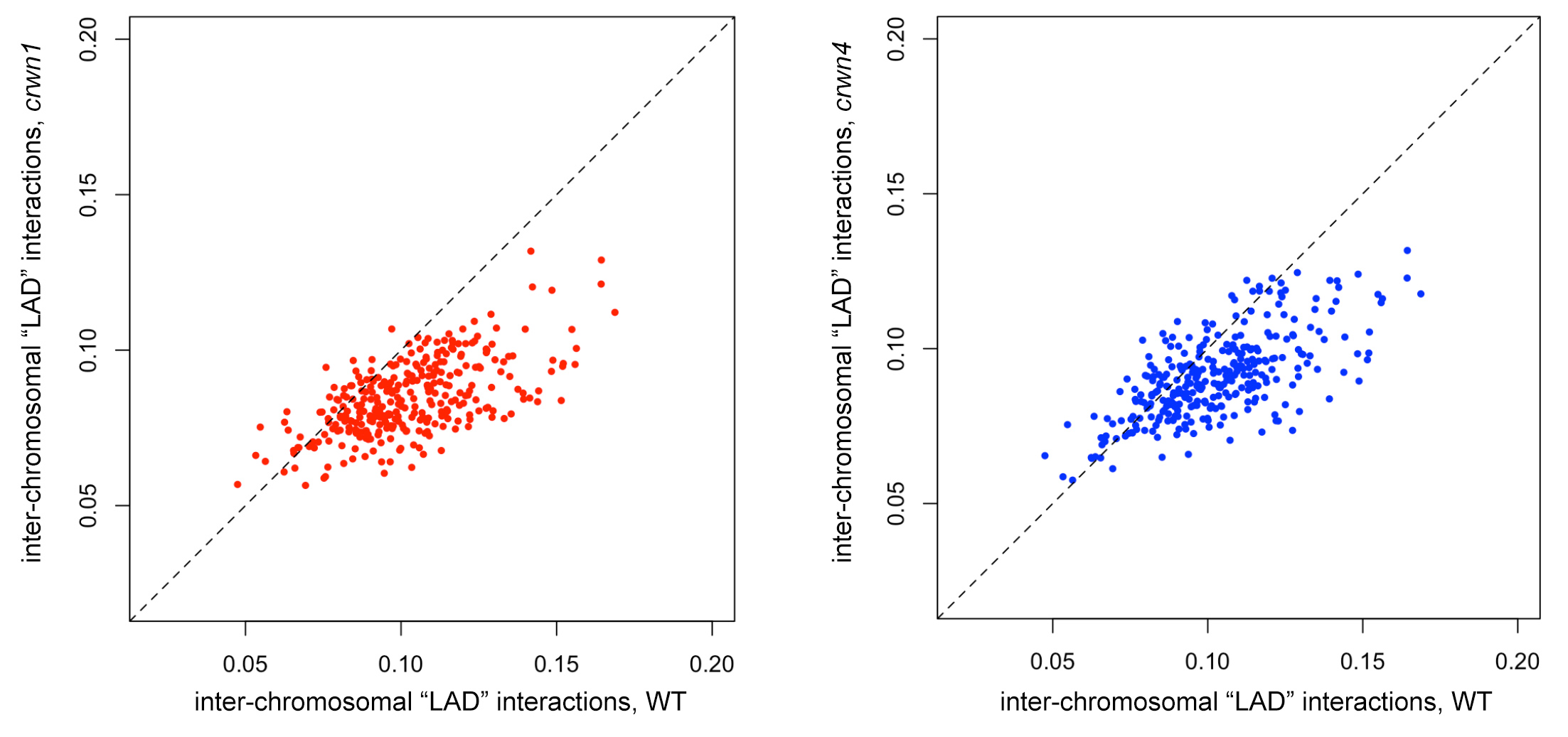
**

**Figure S7. Inter-chromosomal interactions of chromatin regions tethered at the nuclear periphery.**

Inter-chromosomal interactions are according to Hi-C maps (20 kb) shown in Figure S4. Each 20 kb genomic region is annotated as “LAD” or “non-LAD”, depending on whether more than 50 % of this region is covered by NP-enriched domains. For each of these “LAD” genomic regions residing in chromosome arm, its ratio of inter-chromosomal interactions with other “LAD” regions over those with “non-LAD” is computed.

**
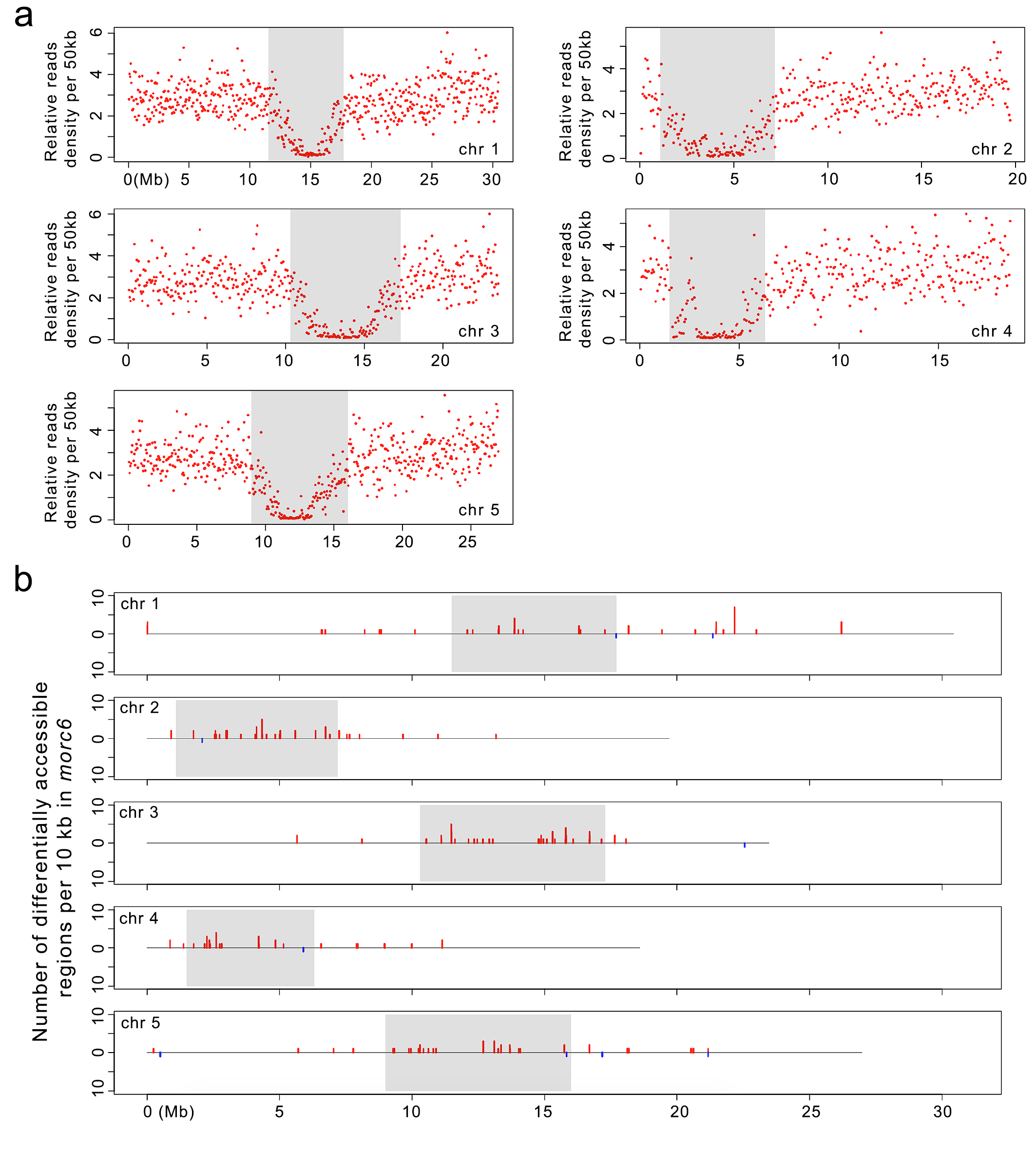
**

**Figure S8. ATAC-seq as a tool to reveal differential chromatin organization patterns and changes in chromatin packing.**

**a.** Relative ATAC-seq reads distribution in 50 kb windows across wild-type *Arabidopsis* 2C nuclei. For biases correction, reads density is normalized against that of a control ATAC-seq experiment using purified genomic DNA as template [2]. Grey blocks depict centromeric and pericentromeric heterochromatin.

**b.** Distribution of differentially accessible chromatin regions in *morc6* 2C nuclei. Red and blue bars denote hyper- and hypo-accessible regions in *morc6*, respectively. Grey blocks depict centromeric and pericentromeric heterochromatin.

**
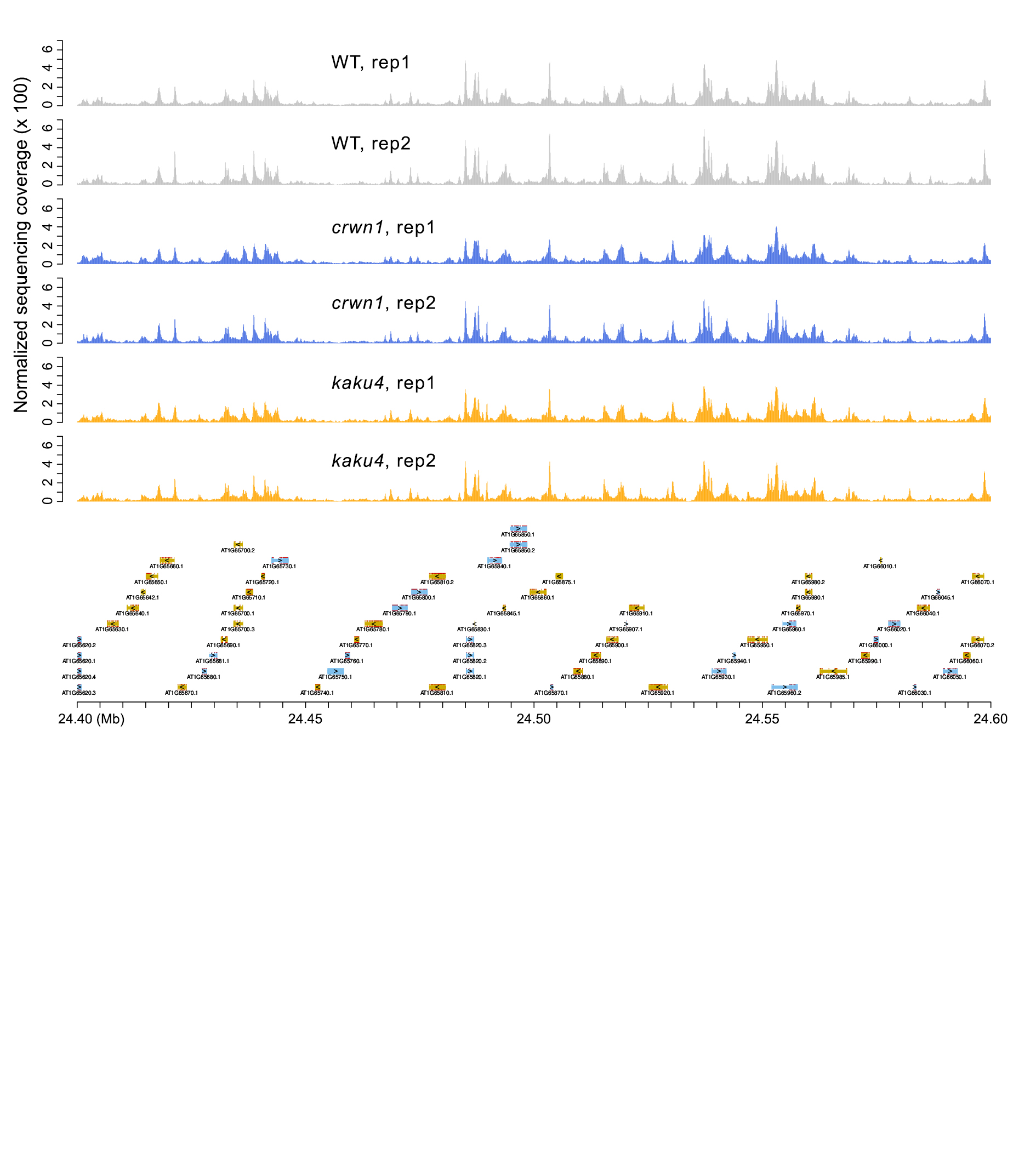
Figure S9. Chromatin accessibility is not affected in *crwn1 or kaku4* mutants.**

A representative genomic region (200 kb) on chromosome 1. ATAC-seq reads density is plotted with 100 bp windows. Note that this region largely overlaps with that targeted by Green probes on chromosome 1 (described in Fig. 1d).

**
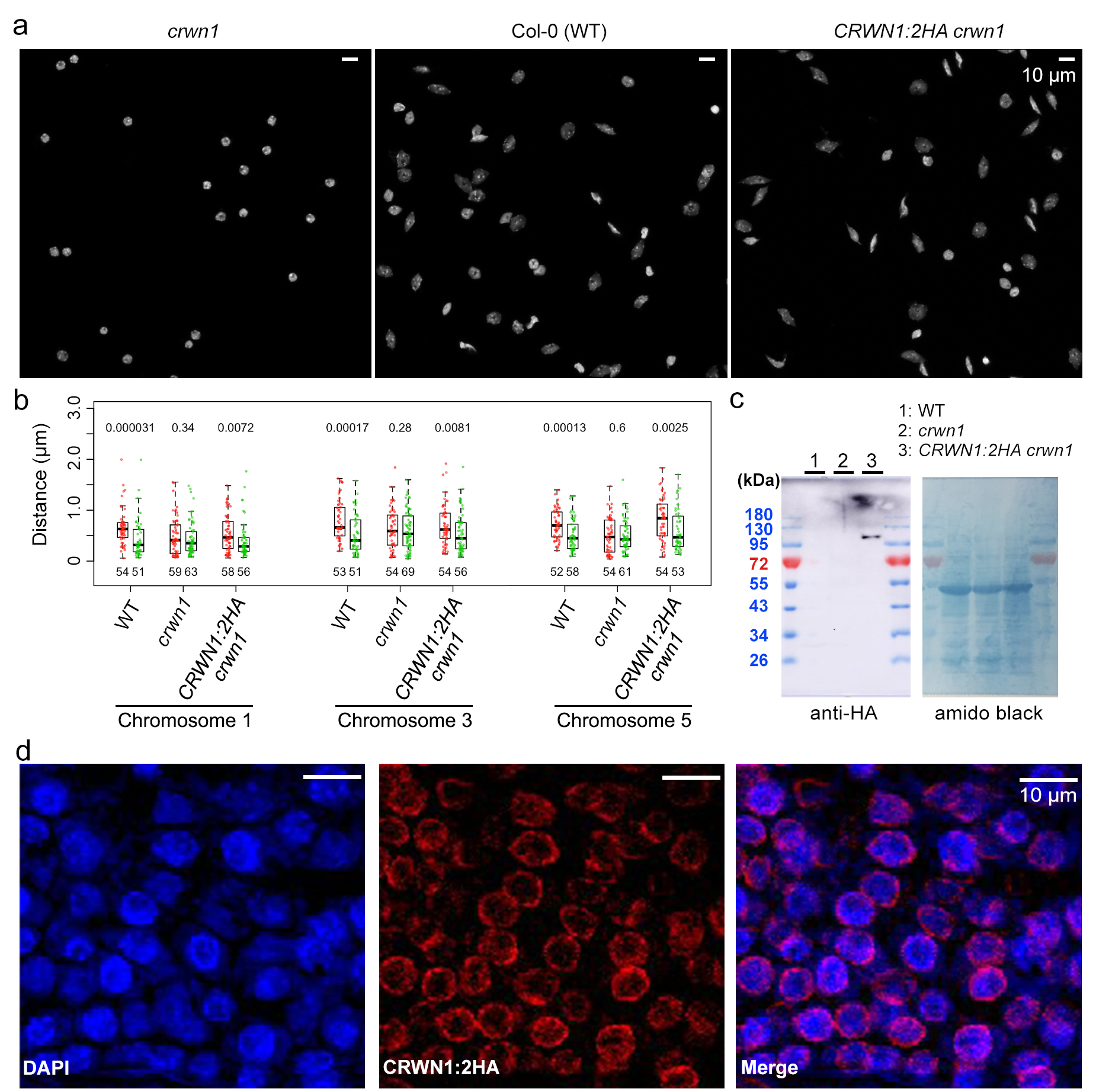
**

**Figure S10. A native CRWN1 tagging construct can fully rescue *crwn1* phenotypes.**

**a.** Comparison of 8C nuclei morphology of different genotypes. The nuclei were isolated from the first true leaf of 2-week old seedlings.

**b.** Distance distribution of probed genomic regions to the NP. Probes are as shown in Fig. 1b. Labels are the same as in Fig. 2.

**c.** Detection of CRWN1:2HA protein in leaf crude extract. The membrane was probed with anti-HA antibody.

**d.** Immunohistostaining of a leaf section of *CRWN1:2HA crwn1* with anti-HA antibodies.


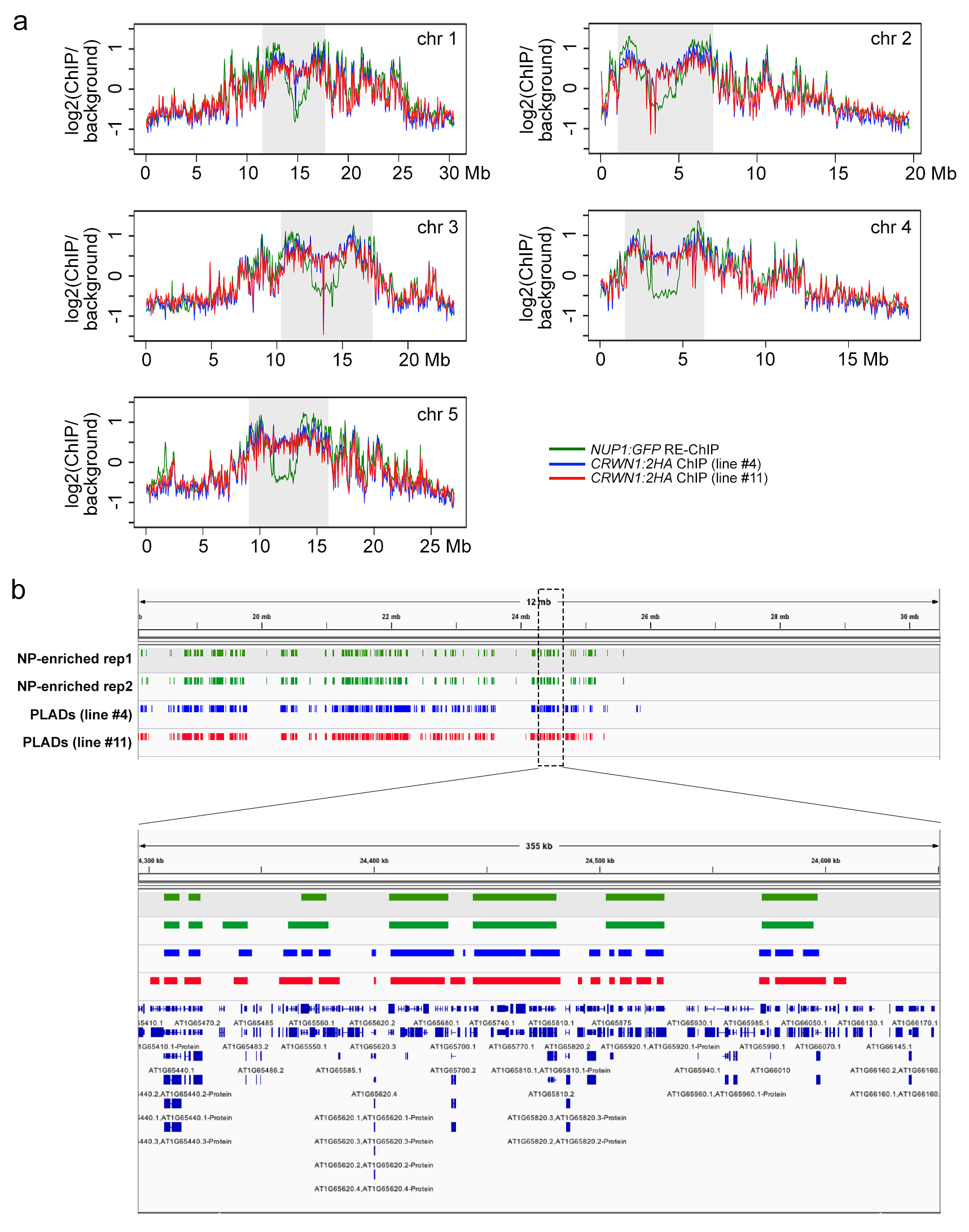


**Figure S11. Comparison of NP-chromatin interaction patterns revealed from different methods.**

**a.** Genome-wide view of *NUP1:GFP* RE-ChIP and *CRWN1:2HA* ChIP signals. The plots are in 50 kb windows. Grey blocks depict centromeric and pericentromeric heterochromatin.

**b.** A zoom-in image of the region shown in Fig. 6c.


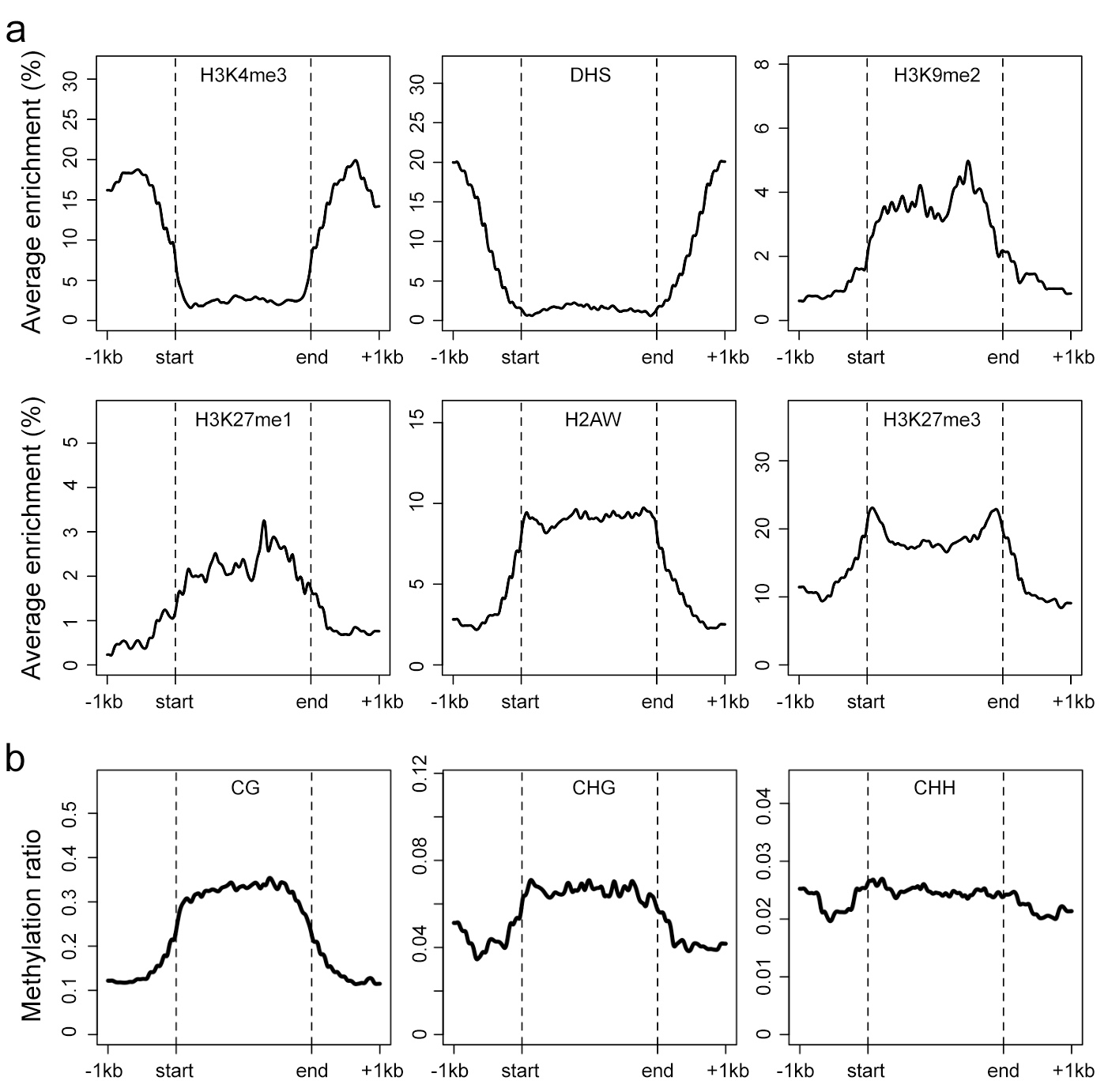


**Figure S12. Features associated with PLADs and their flanking chromatin regions.**

**a,b.** Metagene plots showing histone marks, chromatin accessibility **(a)**, and DNA methylation **(b)** around PLAD regions. Data of the epigenetic marks in **(a)** and DNA methylome in **(b)** are from [3] and [4], respectively. DHS, Deoxyribonuclease I hypersensitive sites. PLADs located in pericentromeric regions are not included. The borders of PLADs are annotated as “start” and “end”.


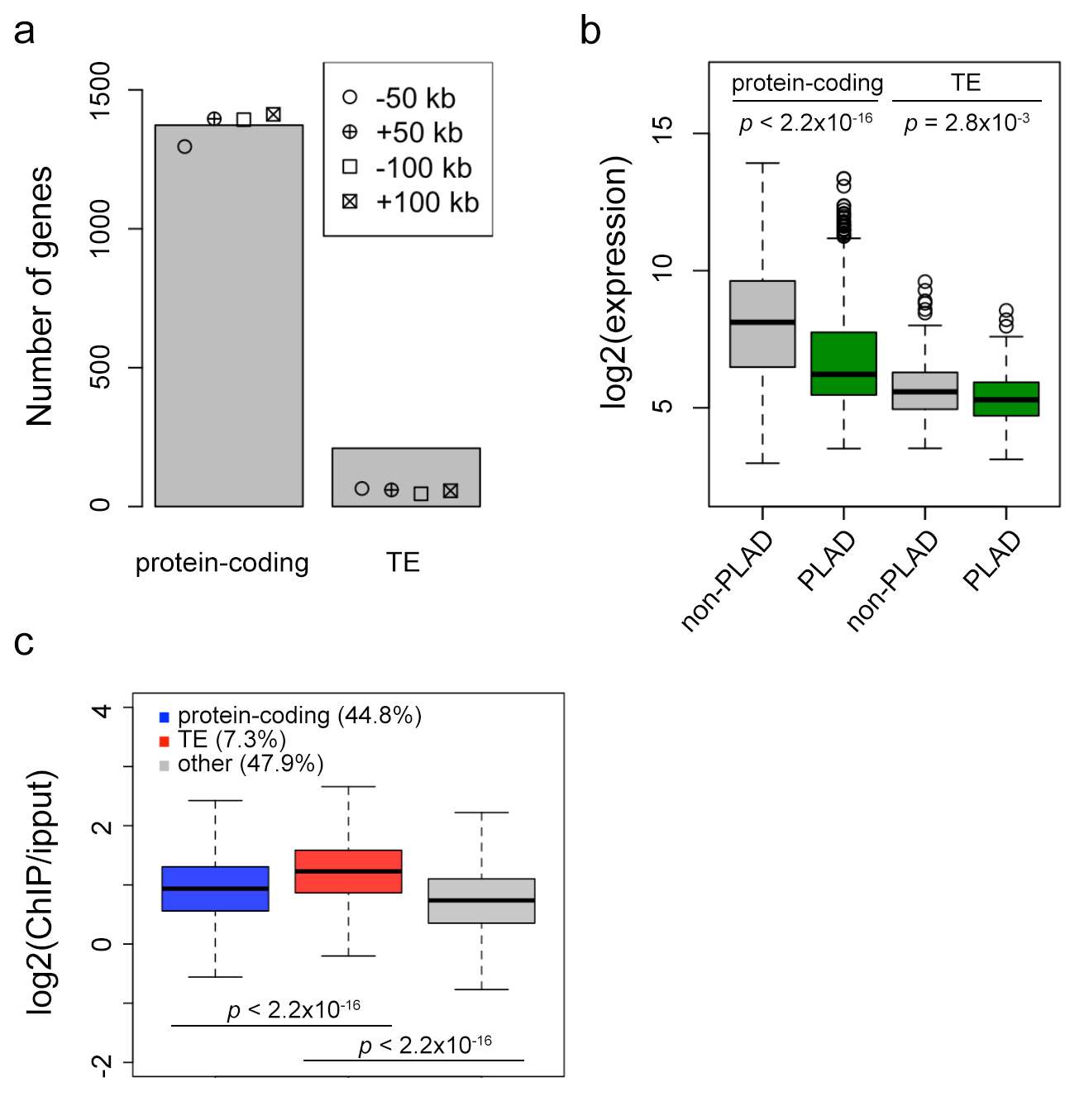


**Figure S13. Genes associated with PLADs.**

**a.** Numbers of protein-coding genes and TEs (transposable elements) residing in PLADs. A gene is considered in PLADs if more than 80 % of its transcribed region overlaps with them. To learn the background number of genes located in PLADs, the PLADs’ coordinates were shifted 50 or 100 kb up- and down-stream, and PLAD genes were annotated again.

**b.** Comparison of gene expression with respect to the PLADs annotation. Gene expression data is from a normalized tilling array dataset [5]. The p-values indicate Mann-Whitney U test results.

**c.** Comparison of CRWN1:2HA ChIP-seq signal in PLADs. Chromatin regions (in 100 bp window) are divided into three groups regarding the genome annotation. The p-values indicate Mann-Whitney U test results.

**
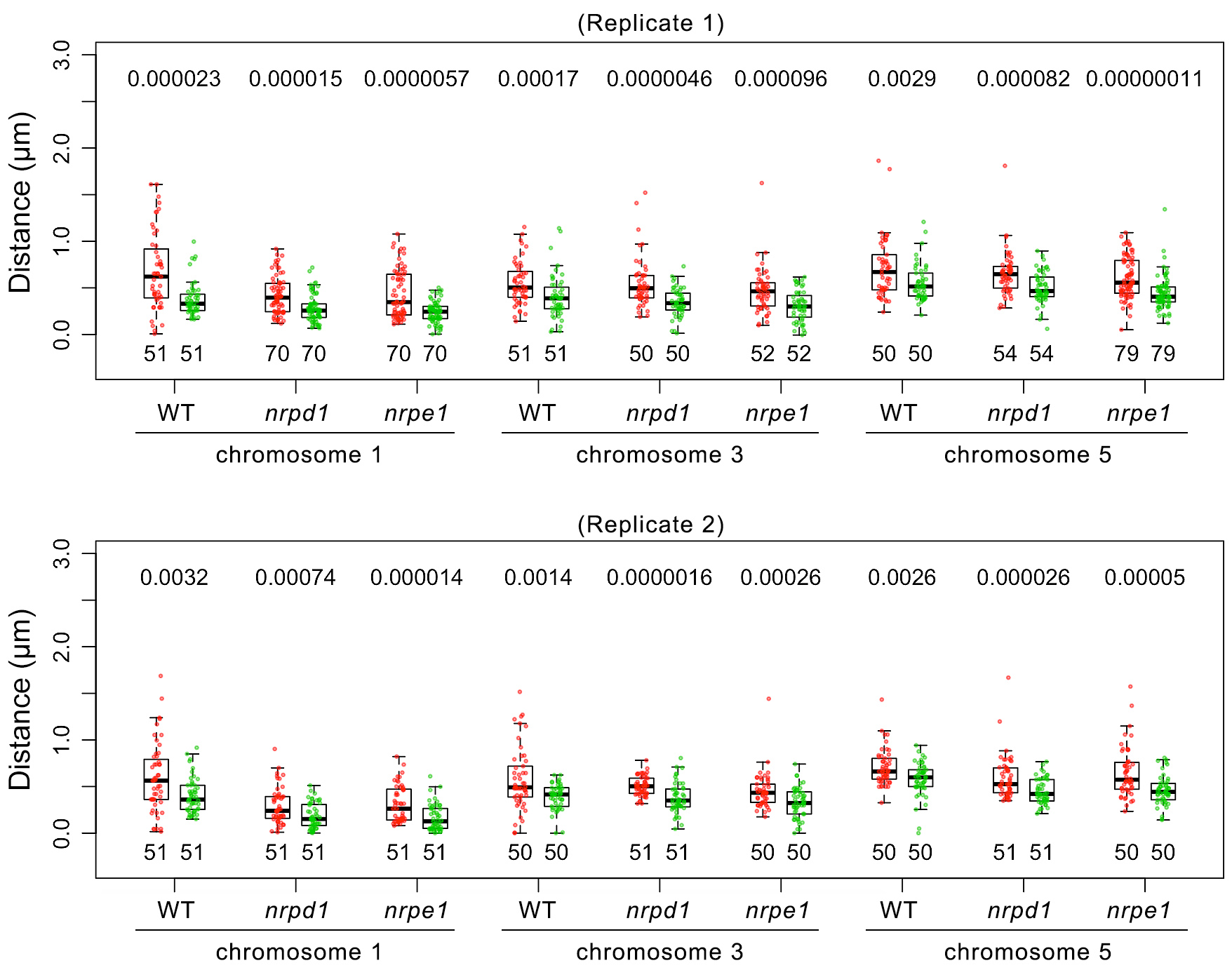
**

**Figure S14. Analyses of FISH signals in RdDM mutants.**

Distances of probed genomic regions to the NP are compared in 2C nuclei. Probes are as shown in Fig. 1d. Labels and annotations are the same as in Fig. 2.


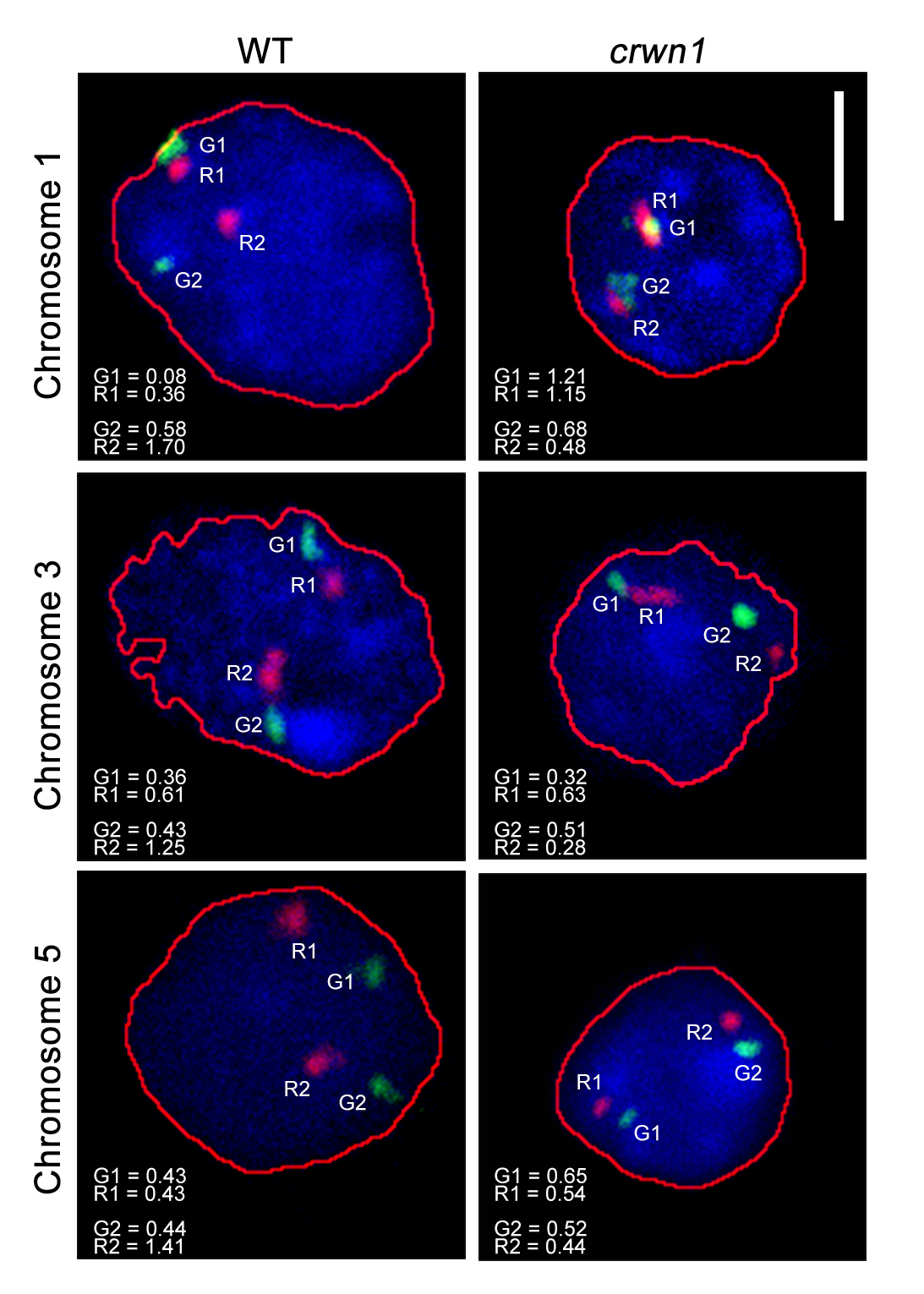


**Figure S15. Representative confocal images showing the localization of probes in WT and *crwn1* 2C nuclei.**

The green and red probes recognizing genomic regions in different chromosomes are as shown in Fig. 1d. Nuclear boundaries were defined in ImageJ with the “ROI manager” tool. Numbers indicate the estimated distances of FISH signal spots to the NP (in micrometer). Scale bar: 2 µm.

**References**

1. Grob S, Schmid MW, Grossniklaus U: **Hi-C analysis in Arabidopsis identifies the KNOT, a structure with similarities to the flamenco locus of Drosophila.** *Mol Cell* 2014, **55:**678-693.

2. Lu Z, Hofmeister BT, Vollmers C, DuBois RM, Schmitz RJ: **Combining ATAC-seq with nuclei sorting for discovery of cis-regulatory regions in plant genomes.** *Nucleic Acids Res* 2017, **45:**e41.

3. Liu C, Wang C, Wang G, Becker C, Zaidem M, Weigel D: **Genome-wide analysis of chromatin packing in Arabidopsis thaliana at single-gene resolution.** *Genome Res* 2016, **26:**1057-1068.

4. Zhu W, Hu B, Becker C, Dogan ES, Berendzen KW, Weigel D, Liu C: **Altered chromatin compaction and histone methylation drive non-additive gene expression in an interspecific Arabidopsis hybrid.** *Genome Biol* 2017, **18:**157.

5. Laubinger S, Zeller G, Henz SR, Sachsenberg T, Widmer CK, Naouar N, Vuylsteke M, Scholkopf B, Ratsch G, Weigel D: **At-TAX: a whole genome tiling array resource for developmental expression analysis and transcript identification in Arabidopsis thaliana.** *Genome Biol* 2008, **9:**R112.
